# Queen quality, performance, and winter survival of imported and domestic honey bee queen stocks

**DOI:** 10.1038/s41598-023-44298-x

**Published:** 2023-10-12

**Authors:** L. A. Holmes, L. P. Ovinge, J. D. Kearns, A. Ibrahim, P. Wolf Veiga, M. M. Guarna, S. F. Pernal, S. E. Hoover

**Affiliations:** 1 Department of Biological Sciences, University of Lethbridge, Lethbridge, AB Canada; 2 Alberta Beekeepers Commission, Edmonton, AB Canada; 3 Agriculture and Agri-Food Canada, Beaverlodge Research Farm, Beaverlodge, AB Canada; 4 National Bee Diagnostics Centre, Northwestern Polytechnic, Beaverlodge, AB Canada

**Keywords:** Entomology, Ecology

## Abstract

Canadian beekeepers have faced high colony mortality each winter over the last decade. Frequently citing “poor queen quality” as a top contributing factor to colony loss, Canadian beekeepers report needing to replace half their queens each year. Domestic queen production exists throughout Canada but is limited due to the short season and can be further limited when colony mortality is high. Consequently, Canadian beekeepers import over 260,000 queens annually, primarily from locations with warmer climates. In this study, newly mated imported queens from Hawaii (USA) and New Zealand were compared to domestic Canadian queens produced in British Columbia; these stocks were evaluated on their morphological and sperm storage characteristics. Stock quality was also evaluated in the field at two locations in Alberta, Canada over two production seasons. Our results show initial variation in queen morphology and fertility among imported and domestic queen stocks. Most striking, the New Zealand queens weighed 10–13% less than the Hawaii and British Columbia queens, respectively upon arrival. Colony performance over a two-year field study suggests: (1) brood pattern solidness has a positive nonlinear correlation with honey production regardless of queen stock and environment; (2) environment (i.e., apiary location) and queen stock variably predict colony health and productivity depending on year; specifically, apiary site appears to be a stronger predictor of colony health and productivity than queen stock in year one, but in year two, queen stock appears to be a stronger predictor than apiary site; (3) high clinical symptoms of chalkbrood may explain the prevalence of poor brood patterns in colonies headed by queens from New Zealand; (4) domestic queens are 25% more likely to survive winter in Alberta than imported queens. Therefore, it is important to consider possible mismatches in disease immunity and climate conditioning of imported queen stocks heading colonies in temperate regions that face drastically different seasonal climates and disease ecology dynamics.

The apiculture industry in Canada continues to expand with increasing demand for hive products and pollination services. In 2022, Alberta had over 300,500 honey bee colonies, representing 39% of honey bee colonies in Canada, and produced 30.4 million pounds of honey valued at 94.7 million CAD^1^. Many Alberta-based colonies also provide pollination services to blueberry or apple production in British Columbia as well as hybrid canola seed production in Southern Alberta. The added value of honey bee pollination to canola seed production was estimated at $6 billion CAD in 2021^2^, representing Canada’s largest pollination market. Importantly, these economic benefits of the beekeeping industry are reliant on the availability of high-quality queens in early spring and summer.

Each honey bee colony is normally headed by a single female queen. Early in life, new queens take one or more mating flights over a few days, then store and use spermatozoa acquired from males (i.e., drones) during mating flights for the remainder of their lives^3^. A queen is typically the mother of all workers in the colony and regulates the colony environment through pheromones and brood production; therefore, queen quality and her mating play pivotal roles in determining colony success and productivity.

Beekeepers throughout Canada frequently cite “poor queen quality” as a top contributing factor to colony mortality^4^. Poor quality queens are often described by beekeepers as short-lived, easily superseded, or having poor oviposition (e.g., a drone layer). Poor queen quality can also contribute to sub-lethal losses in colony productivity and therefore, beekeepers report needing to replace half their queens each year^5^. Beekeepers replace queens either proactively, introducing a new young queen to a colony to replace an older queen, or as required to replace failing or lost queens. Queens are generally replaced in the spring in Alberta (i.e., April through June), although some queens are also replaced later in the season, as needed.

Beekeepers have two options for obtaining new queens: they can either rear their own virgin queens and then provide them with the opportunity to mate, or purchase mated queens from commercial queen breeders. Domestic queen production exists throughout Canada but is limited because queen rearing and mating requires reliable summer conditions and the availability of mature drones, which are in short supply in early spring. As a result, Canadian beekeepers import over 260,000 queens annually worth $8.5 million CDN^2,6^. Government of Alberta^7^ surveys show that 99 percent of purchased queens in Alberta in 2021 were imported from the warmer parts of the USA (96.4%), including, Hawaii and California, or from contra-season countries in the southern hemisphere (< 1.4%) (e.g., New Zealand, Australia, and Chile). Only 2% of purchased queens in Alberta in 2021 were purchased from Canadian queen breeders (e.g., British Columbia and Saskatchewan (i.e., 1%) and Alberta (i.e., 1%)^7^. As a result, imported queens that are bred in warm climates are heading colonies in Canada where they are subjected to harsh winter conditions.

While beekeepers in Canada are aware that high quality queens are intrinsic to colony health and productivity, they currently have insufficient information to support their management decisions. In addition, there are no standard criteria or quality control measures in place for beekeepers to evaluate their queens for quality at shipment, or their performance in colonies. Therefore, the goal of this project was to characterize the variability of queen quality traits that exists among some commonly imported and domestic queen stocks^8,9^, relate such trait measurements to colony productivity in the field, and to provide data to beekeepers to support beekeeping management decisions as they pertain to queen quality.

## Methods

### Establishing experimental apiaries

Stock assessment apiaries were established in May of 2017 at three sites: one in Beaverlodge, Alberta managed by Agriculture and Agri-Food Canada researchers (hereafter referred to as “North”, 55.201904, −119.396172), and two near Lethbridge, Alberta managed by researchers (“South RS”,49.711172, −112.700033), and by a participating beekeeper (“South BK”, 49.726262, −112.656043). The South BK colonies were moved within 50 km of Lethbridge as necessary to pollinate hybrid seed canola in both 2017 and 2018 (50.209861, −112.603773 and 50.230140, −112.478519, respectively).

At each site, 32 (South BK), 37 (South RS) and 60 (North) small queenless colonies (“splits”) were established in nine frame brood chambers to start the experiment, each with similar comb composition (i.e., honey, pollen, and empty comb) and populations of adult bees and brood. Specifically, three frames of brood with their attending adult bees were added to each split and an equivalent of two frames of bees were shaken into each split. Colonies were maintained in single brood chambers and managed according to normal beekeeping practices for the region. Pre-weighed honey supers were added to each colony as needed throughout the honey flow each season. Supplemental feeding was provided with pollen patties and sucrose syrup at establishment and prior to winter.

In fall 2017, South RS and North colonies were treated for *Vairimorpha sp.* (*Nosema*) and *Varroa* with Fumagilin-B™ and Apivar®, respectively, following label instructions. South BK colonies were not treated for *Vairimorpha* in fall 2017 but were treated for *Varroa* with oxalic acid sublimation in compliance with the typical management practices of the participating beekeeper. All colonies were overwintered indoors at 4° C between first week of November 2017 and third week of April 2018.

### Imported and domestic stock queens

Newly mated domestic and imported queen stocks arrived from British Columbia (BC), Hawaii (HI), and New Zealand (NZ) between May 16th and June 2nd, 2017. Domestic queens from BC were mixed race, Hawaiian queens were “carniolan”, and New Zealand queens were “carniolan”. Queens were marked with a unique colour for each stock and kept in a queen bank for up to 17 days until their introduction to experimental colonies. A subset of 15 queens from each stock were destructively evaluated upon arrival at the National Bee Diagnostics Centre (NBDC) for queen morphological characteristics, sperm counts, and sperm viability. Remaining queens were introduced to the queenless splits across all sites (*N* = 129) on June 1–2, 2017. During the following week, any queens not liberated from their shipping cages by the workers were manually released and adult bee populations were equalized to account for worker drift among colonies.

### Characterizing queen quality

Queen morphological measurements (e.g., head width, thorax width and length, body weight), spermatheca diameter, sperm count and viability, ovary weight, and ovariole counts were completed on a subset of 15 queens from each stock (i.e., BC, HI, and NZ) upon arrival in spring 2017 (described below). Upon experiment completion in August 2018, remaining experimental stock queens (15 North, 12 South BK, and 2 South RS) were sent to NBDC for sperm count and viability, however, due to concerns over shipping older queens long distances, sperm viability was not analyzed for queens from the two Southern sites.

To evaluate metrics of queen quality, each queen was temporarily immobilized in a freezer and then its fresh weight was recorded to the nearest 0.1mg on an analytical balance (CPA 224S, Sartorius, Goettingen, Germany). Thorax length, thorax width, and head width were measured to within 0.1mm using digital calipers, and then each queen was sacrificed by decapitation^8^. Each queen’s abdomen was dissected while being viewed through a stereomicroscope (Model SMZ1000, Nikon) and her spermatheca and ovaries were removed. Spermathecal diameter without tracheal netting was measured using a calibrated ocular micrometer in the dissecting stereomicroscope.

Immediately after isolating and measuring each spermatheca, each was ruptured in 0.5 ml of Buffer D^10^ in a glass vial. Fifteen µl of SYBR 14 and 8µl of propidium iodide were added to each vial and incubated at room temperature for 15 min (adapted from LIVE/DEAD Sperm Viability Kit, Molecular Probes, Eugene, OR, USA). Stained spermatozoa were immediately loaded onto a 0.02 mm cell depth Thoma counting chamber (Hawksley and Sons Ltd, Lancing, Sussex, UK) and both live and dead sperm were visualized and counted using a fluorescence microscope (Fluoview FV10C-W3, Olympus, Tokyo, Japan). Live sperm fluoresce at green wavelengths (e.g., EM 520–570 nm), while dead sperm fluoresce at red wavelengths (e.g., EM 650 nm). Average sperm count was corrected for dilution.

Right ovaries (defined as the ovary on the right side of the queen pinned ventral side down) were carefully removed with forceps and transferred immediately to a slide. Ovary wet weight was measured to the nearest 0.1mg. The number of ovarioles per ovary was immediately determined by visual counting under a stereomicroscope using a fine needle to isolate individual ovarioles.

### Colony level performance parameters

Colony performance parameters including brood pattern, hygienic and defensive behaviour, honey production, and capped brood and adult bee population sizes were recorded for each colony in 2017 and 2018 (described below). We also recorded any observations of brood disease and failing queens and sampled each colony for *Varroa* and *Vairimorpha* three times between June and October in 2017, and again in April and July in 2018. Colonies were removed from the experiment if they were unproductive (i.e., less than four frames of bees) or if the original queen was no longer present.

Sealed brood pattern was measured by evaluating the solidness of the pattern of capped brood in each colony using a rhombus-shaped cut-out. A rhomboid shape encompassing 100 cells (10 × 10) was cut out of corrugated plastic and overlaid on a patch of capped brood. Patches of capped brood with the greatest amount of capped brood continuity were selected, and four patches of capped brood were evaluated per colony on at least two separate frames. Brood pattern solidness was calculated as the number of cells not containing capped brood subtracted from 100. Brood solidness measurements began once capped brood was present after queen introductions and were repeated approximately every two weeks between June and August in both study years for a total of 8–10 assessments per colony. Brood pattern solidness measurements were averaged for each colony within each year.

To monitor disease pressure and determine any differences in disease tolerance among stock queens, colonies were repeatedly sampled for *Vairimorpha* and *Varroa*. We followed Fries et al.^11^ and Human et al.^12^ for all *Vairimorpha* sampling, collecting approximately 100 bees from honey frames. Techniques of Gatien and Currie^13^ were followed for all *Varroa* sampling, collecting approximately 300 bees from brood nest frames. We sampled *Vairimorpha* and *Varroa* in mid-June 2017 to establish disease load of each colony prior to population turnover, and then again in mid-August and mid-October. Sampling bees for *Vairimorpha* and *Varroa* resumed in April 2018, and again at the end of the experiment in July 2018. *Vairimorpha* and *Varroa* counts were averaged for each colony within each sampling year. Other brood diseases including American foulbrood, European foulbrood, chalkbrood, and sacbrood were also monitored for presence or absence. However, after noting the presence of chalkbrood in several colonies at the South RS site in late summer 2017, the number of brood cells showing clinical symptoms of chalkbrood were counted for each brood frame examined during the solid brood pattern assays at the South sites.

A freeze-killed brood assay^14,15^ was used to characterize hygienic behaviour of each stock. One frame with a solid patch of capped pupal brood (identified by their pink or purple eyes) was selected per colony. Two PVC pipes (6 cm outer diameter and 5 cm inner diameter, cut 15 cm long) were pressed into the brood comb and filled with approximately 300 ml of liquid nitrogen to freeze the brood. PVC tubes were removed once brood thawed, and the number of brood cells was calculated by subtracting the number of empty brood cells and cells containing nectar and honey from the number of cells in the patch. The frame was then returned to the brood nest and checked 24 h later (+ /- 5 min) for the number of capped brood cells that had been removed (i.e., cleaned out) by workers. Two hygienic behaviour assays were performed one week apart in mid-August 2017 and an additional two in June of 2018. A hygienic behaviour score was recorded for each colony as the percent of dead brood removed by the colony within a patch within 24 h. Hygienic behaviour scores were averaged across the two assays within each sampling year.

Defensive behaviour assays adapted from Guzman-Novoa et al.^16,17^ were performed after completing the hygienic behaviour assays on the same day. A black suede leather patch (7.6 cm × 7.6 cm) suspended from a pole with white string was gently waved 5 cm above the brood chamber of each colony for 2 min and the number of stings on the patch was recorded. Colonies on the same pallet were tested simultaneously. Defensive behaviour scores were averaged across two assays within each sampling year.

The weight of honey produced by each colony was measured in 2017 and 2018. Pre-weighed honey supers were removed throughout the honey flow and their full weights recorded. Honey production for each colony collected in late July and August was summed for each sampling year.

In early August of 2017 and July of 2018, all the frames containing bees in each colony were removed and photographed in the early morning before bee flight. To determine the population of adult bees, we used the photo ladder method described by Ovinge and Hoover^18^, where the adult bee photos were viewed on a computer, and compared to a wall of representative photos to estimate how many bees were in each photo. A 10% subset of 2017 photos and a 7% subset of 2018 photos were manually counted to validate our photo ladder method. R^2^ values were 0.97 and 0.94 for the 2017 and 2018 manual photo counts, respectively.

After pictures of adult bees were taken, bees on frames containing capped brood were carefully brushed off to photograph capped brood cells. These photos were analysed with HoneybeeComplete software (version 5.4 WSC Scientific, Heidelberg, Germany) to estimate the number of capped brood cells per frame side. Frame sides of capped worker brood were summed for each colony within each year.

Bee cluster size of each colony were recorded in October 2017 and in late April 2018. Bee cluster size was measured by viewing the top and bottom of each colony’s brood box and visually estimating the number of inter-frame spaces filled with bees^19^. The total number of inter-frame spaces from top and bottom views was averaged.

### Statistical analyses

All statistical analyses were done using the R software environment version 4.2.2^20^.

A principal component analysis (PCA) was done on all queen morphological traits, including weight, head width, thorax width and length, spermatheca diameter, ovariole count, ovary weight, sperm count, and sperm viability using the *prcomp* function in the R stats package^20^ with scale standardization (i.e., scale = TRUE) to account for different measurement units across variables. Observations for queen stocks (e.g., BC, HI, and NZ) were plotted in two-dimensional space to visualize their distribution as defined by the two principal component axes that explain the largest variation among queen morphological traits.

We used generalized linear models (GLMs), and quasi generalized linear models where appropriate, to characterize queens’ morphological traits including, mass, head width, thorax length and width, spermatheca volume, sperm count and viability, the number of ovarioles per ovary, and ovary mass across the categorical variable queen stock source (i.e., BC, HI, and NZ using the *mgcv* package^21,22^. Statistical significance was evaluated using model selection^22–24^ with Akaike Information Criteria (AIC) and quasi-AIC, where appropriate. The general linear hypothesis testing function *glht* in the Multcomp package^25^ was used for post-hoc analyses of GLMs, where applicable; p values were adjusted for Type I error using the Bonferroni method.

A generalized linear model was used to perform a logistic regression between queen sperm count and sperm viability across the categorical variable queen stock on the 45 queens sampled at the start of the project (i.e., 15 queens per stock) and on the 13 queens recovered from the North site at the end of the project in 2018. Sperm viability was estimated from the number of living and dead sperm and expressed as a proportion of viable sperm.

Generalized linear models and AIC model selection were also used to characterize mean hygienic behaviour scores, defensive behaviour scores, mean solid brood pattern scores, mean honey yields, mean *Vairimorpha sp.* and *Varroa* counts, queen introduction success, overwintering survival, overall survival, chalkbrood presence/absence and abundances, and bee cluster sizes, across categorical variables, queen stock source, apiary site (e.g., North, South RS, and South BK), and year (e.g., 2017 and 2018) (Table S1). We used a 42-day period to assess queen introductory success, (i.e., the length of two brood cycles). Winter colony losses were analyzed for the number of colonies alive in fall 2017. Colonies that did not survive or failed to maintain at least four frames of bees by May 21^st^, 2018^4^, were characterized as winter colony losses and pulled from the experiment.

Model selection for our winter colony losses included one model within two delta AIC units of the top model. Thus, we performed nonparametric bootstrapping to infer statistical significance on the top model. We created a sampling distribution of mean winter colony survivorship for each queen stock by resampling the experimental data with replacement 20,000 times. Mean and confidence intervals were estimated from the bootstrapped sampling distribution for each queen stock using Efron’s^26^ percentile method. We created a null distribution of mean winter colony survivorship by randomly assigning a queen stock treatment to each bootstrapped statistic mean of our sampling distribution. The probability of observing differences in winter colony survival among queen stocks was calculated from a null distribution of differences of mean colony survivorship using the percentile method^26^ (Fig. S1).

A principal components analysis^20^ was also done on colony level health and productivity assay variables, including *Varroa* mite load, *Vairimorpha* spore load, solid brood pattern score, cluster score, honey production, and hygienic and defensive behavioural scores across years of sampling. Observations were plotted in two-dimensional space to visualize their distributions as defined by the two principal component axes that explain the largest variation among the colony level health and productivity variables for both queen stocks (e.g., BC, HI, and NZ) and apiary site (e.g., North, South BK, and South RS).

We also used generalized linear models and AIC model selection to perform four analyses of covariance between solid brood pattern score and hygienic behaviour score, the number of capped brood, and the number of adult bees across the categorical variable queen stock (e.g., BC, HI, and NZ) and apiary site (e.g., North, South BK, and South RS). We also performed two analyses of covariance between chalkbrood abundance and solid brood pattern, and hygienic behaviour score across the categorical variables queen stock and South apiary sites (e.g., South BK and South RS). Finally, we performed a generalized linear model with AIC model selection to characterize honey production across solid brood pattern scores. The model was well characterized by an under-dispersed Gamma with an inverse link error distribution.

## Results

### Characterizing queen quality

**Figure 1:**
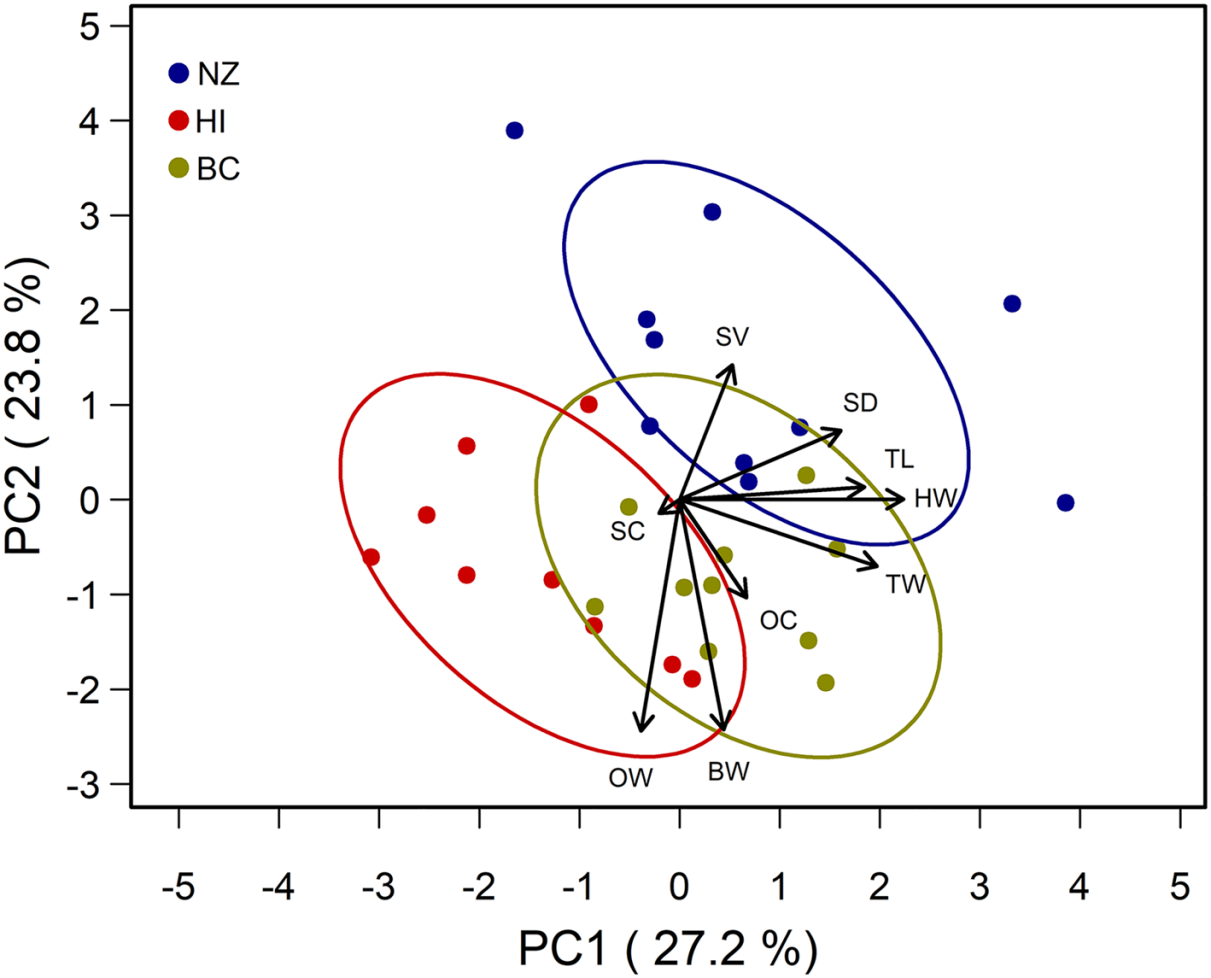
Principal component analysis on queen morphological traits including thorax length (TL), thorax width (TW), head width (HW), body weight (BW), ovariole count (OC), ovary weight (OW), sperm count (SC), spermatheca diameter (SD), and sperm viability (SV) from three queen stock sources, British Columbia (BC), Hawaii (HI), and New Zealand (NZ) in yellow, red, and blue, respectively. The variation in queen morphological traits explained by principal components one and two are indicated in parentheses. The figure shows that head width, thorax width, thorax length, and spermatheca diameter were most correlated with PC1. Body weight and ovary weight were most correlated with PC2, and sperm count was most correlated with PC3, which explained 17% of the variation of queen morphological traits measured.

Differences in queen morphology among the stocks were found (Tables 1 and S1). In general, HI queens were smaller than BC and NZ queens in head, thorax, and spermathecae size, but NZ queens weighed significantly less overall with smaller ovaries than HI and BC queens. Principal components (PC) one and two explained 27.2% and 23.8% of the variation in queen morphological traits measured, respectively (Fig. 1). Head width, thorax width, thorax length, and spermatheca diameter were most correlated with PC1. Body weight and ovary weight were most correlated with PC2, and sperm count and ovariole count were most correlated with PC3, explaining 17% of the variation of queen morphological traits measured. The biplot of PC1 and PC2 shows separation of the three queen stock populations. HI queens separate from BC and NZ queens along the PC1 axis, while HI and BC queens separate from NZ queens along the PC2 axis.

**Table 1:** Characterizing queen honey bee morphologies for each queen stock (BC = British Columbia, HI = Hawaii, NZ = New Zealand) in June 2017. Within each row, means followed by different letters are significantly different (*p* < 0.05) after performing general linear hypothesis post-hoc testing on the top model selected by Akaike Information Criterion (AIC) or quasi-AIC where appropriate (Table S1) *p* values were adjusted for Type I error using the Bonferroni method.

Morphological trait	Mean ± SE		
	BC	HI	NZ
N	15	15	15
Weight (mg)	209.47 ± 3.68 a	203.4 ± 3.51 a	183.31 ± 3.51 b
Head width (mm)	3.8 ± 0.02 a	3.65 ± 0.03 b	3.79 ± 0.05 a
Thorax length (mm)	4.88 ± 0.05 ab	4.73 ± 0.04 b	4.96 ± 0.07 a
Thorax width (mm)	4.72 ± 0.02 a	4.47 ± 0.04 b	4.59 ± 0.04 b
Spermatheca Volume (mm^3^)	0.79 ± 0.02 a	0.67 ± 0.02 b	0.84 ± 0.02 a
N	14	15	15
Sperm Count (millions)	6.48 ± 0.44 a	5.93 ± 0.27 a	5.3 ± 0.37 a
Sperm Viability (%)	84.57 ± 1.68 ab	83.3 ± 1.57 b	89.13 ± 1.82 a
N	10	10	10
No. of Ovarioles per Ovary	157.2 ± 5.26 a	145.7 ± 5.83 a	148.2 ± 5.65 a
Ovary wet weight (mg)	21.78 ± 0.74 a	22.91 ± 0.97 a	16.24 ± 1.4 b

*N refers to the number of queens sampled from each stock population (i.e., BC, HI, and NZ).

The number of ovarioles per ovary did not differ among queen stocks (Tables 1 and S1). Ovary weight, however, did differ among queen stocks, with NZ queen ovaries 29–34% lighter than HI or BC queens, respectively, and HI and BC queen ovaries similar in weight (Table 1). Sperm count (i.e., the number of spermatozoa stored in a queen’s spermathecae) did not differ among queen stocks (Tables 1 and S1). Sperm count varied from 3.1 to 10.4 million with an average of 5.9 million stored sperm per queen, regardless of queen stock. There were, however, small differences in sperm viability among stocks. The NZ queens had 6.7% higher sperm viability than HI queens, but sperm viability did not differ among the BC and HI queens or among the NZ and BC queens (Table 1). We found no significant correlation among sperm count and viability for queens assayed at the start of the experiment (Table S2).

End-of-life queen sperm count data was only available for a subset of queens that survived and were successfully located at the end of the experiment in July 2018 (*N* = 28). Because there were only two queens recovered from the South RS site, and end-of-life sperm count did not differ among these two queens and the 11 queens recovered from the South BK site, we pooled the queens from the two South sites. The best model for predicting end-of-life sperm count included the independent effect of site (e.g., North, and South), where queens from North sites (3.47 ± 0.61 million) had 44.47% lower end-of-life sperm counts than queens from South sites (5.45 ± 0.33 million) (Table S3).

End-of-life sperm viability was not characterized across the North and South sites since queens from the South sites were not analysed for sperm viability for fear transit to the lab in northern Alberta would affect viability. Within the North site, end-of-life sperm viability was not different among queen stocks (F_2,12_ = 1.21, *p* = 0.332), however, we did find a positive correlation between sperm viability and sperm count for these North site queens (*r* = 0.312; df = 13, *p* = 0.001).

### Colony level performance parameters

**Figure 2:**
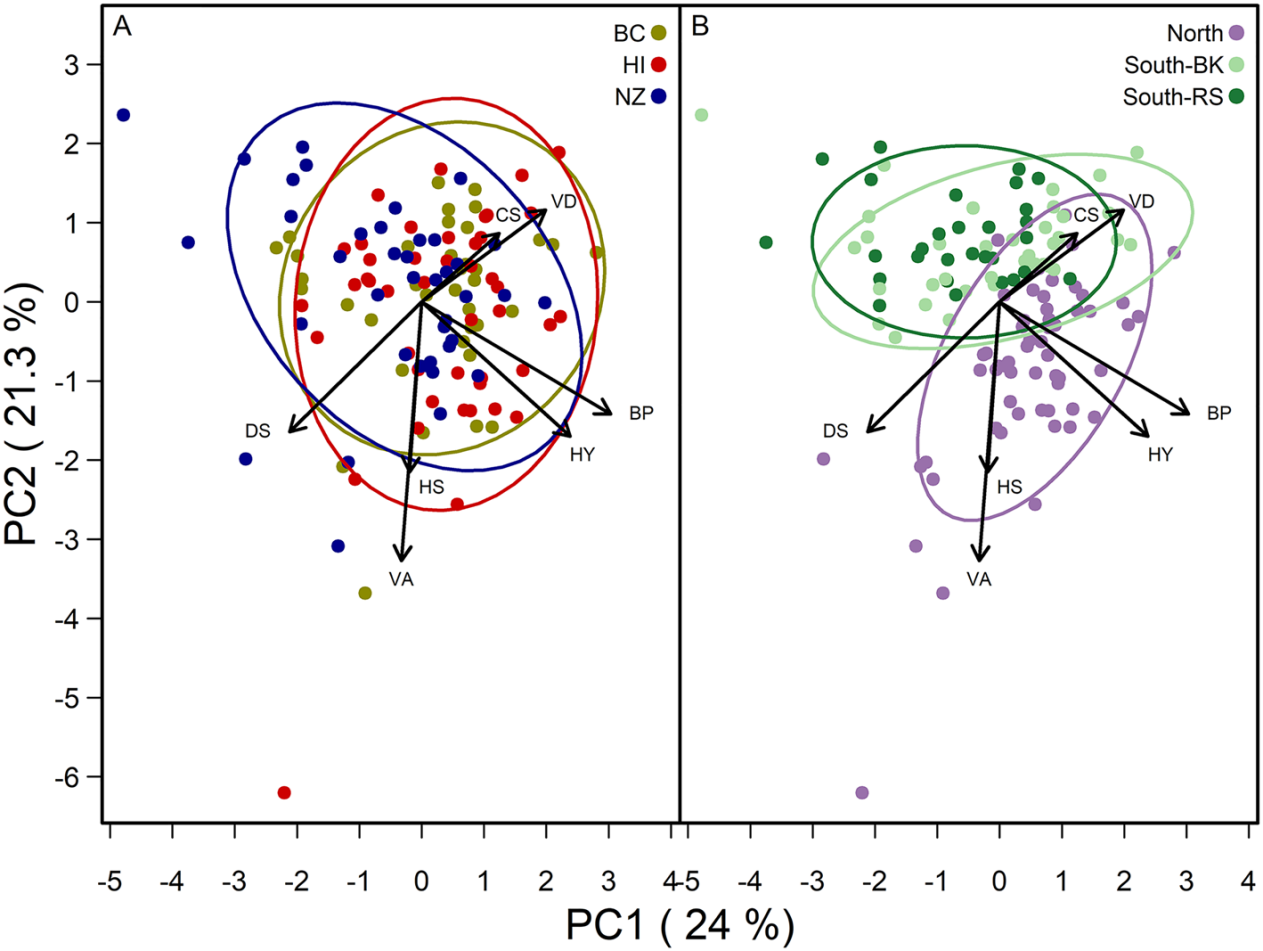
Principal component analysis on colony level performance parameters including *Varroa destructor* mite load (VD), *Vairimorpha* spore load (VA), cluster Score (CS), honey (HY) production, brood pattern (BP), hygienic score (HS), and defensive score (DS) across (**A**) three queen stocks from British Columbia (BC), Hawaii (HI), and New Zealand (NZ) in yellow, red, and blue, respectively, and (**B**) three apiary sites, North (i.e., Beaverlodge, Alberta), and South BK and South RS (i.e., Lethbridge, Alberta) in purple, light green, and dark green, respectively. The variation in colony level performance parameters explained by principal components one and two are indicated in parentheses. The figure shows that honey yield, defensive behaviour, and solid brood pattern were most correlated with PC1, and *Vairimorpha sp.* spore load and hygienic score were most correlated with PC2.

Apiary site influenced colony performance more than queen stock as the biplot of PC1 and PC2 shows variation among colony performance parameters separates more by apiary location (i.e., North, South BK, and South RS) than by queen stock (Fig. 2). Specifically, North colonies separate from both South locations along PC1 where solid brood pattern score, honey production, and defensive score correlate most strongly and along PC2 where *Vairimorpha* and hygienic score correlate most strongly (Fig. 2b). Principal components one and two explained 45% of the variation in colony health and productivity parameters measured. Details on the specific colony performance parameters are presented below.

#### Hygienic and defensive behaviour

Hygienic behaviour and defensive behaviour scores differed among apiary sites, but differences depended on sampling year (Tables S4 and S5, respectively). Specifically, in 2017, colonies from the South RS site (73.13 ± 3.16%) had higher hygienic scores than colonies from the North (65.54 ± 2.29%) and South BK (64.15 ± 3.07%) (Table S6). However, in 2018, North (80.30 ± 2.15%) colonies were 20% and 40% more hygienic than South BK (65.82 ± 3.68%) and South RS (53.59 ± 3.49%) colonies, respectively, increasing 20% for North colonies, but decreasing 30% for South RS colonies (Table S6). Defensive behaviour did not differ among sites in 2017 (Mean = 1.75 ± 1.90), however, South BK (14.78 ± 2.34) and North (15.94 ± 1.88) colonies in 2018 had 159% higher defensive scores than colonies from all sites in 2017, and 107% higher defensive scores than the South RS (4.63 ± 2.42) colonies in 2018 (Table S6). Queen stock was not included in the top model for predicting hygienic behaviour or defensive behaviour (Tables S4 and S5, respectively).

#### Health and productivity

Pre- and post-population turnover levels of *Varroa* were not significantly different for colonies headed by different queen stocks within each apiary site (F_2,289_ = 2.58, *p* = 0.08) and therefore *Varroa* counts for each colony were averaged across all three samples in 2017 and two samples in 2018. *Varroa* counts differed across sampling year (Table S7) but did not differ among apiary sites or queen stocks. *Varroa* was 171% higher overall in 2017 (Mean = 0.63 ± 0.05 mites/100 bees) compared to 2018 (Mean = 0.05 ± 0.07 mites/100 bees).

The best model for predicting *Vairimorpha* spore counts included an interaction term between sampling year and site plus an interaction term between site and queen stock (Table S8). *Vairimorpha* spore counts did not differ between sampling years or among queen stocks for North site colonies (Fig. S2). However, we did find differences among the southern apiary sites across sampling years and among queen stocks (Fig. S2 and Table S8). In 2017, north colonies had higher *Vairimorpha* spore counts than the two south apiaries, however, in 2018, we found larger differences in *Vairimorpha* spore counts among all apiary sites (Fig. S2). Colonies headed by HI queens had lower *Vairimorpha* spore loads than colonies headed by BC or NZ queens at the South BK site, and colonies headed by BC queens had higher spore loads than colonies headed by HI and NZ queens at the South RS site (Fig. S2).

The best model for predicting the presence of chalkbrood at the Lethbridge apiaries included apiary site and sampling year (Table S9). The proportion of colonies showing clinical signs of chalkbrood was higher at the South RS apiary compared to the South BK apiary (*Z* = 4.47, *p* < 0.001) regardless of sampling year. However, the presence of chalkbrood increased only slightly across sampling years regardless of apiary site (*Z* = 2.20, *p* = 0.056). In 2017, chalkbrood was present in 4% of colonies at the South BK apiary and 57% of colonies at the South RS apiary. In 2018, the proportion of colonies positive for chalkbrood increased to 23% of colonies at the South BK apiary and 80% of colonies at the South RS apiary. In colonies positive for chalkbrood at the South apiary locations, the best model for predicting the number of brood cells showing clinical signs of chalkbrood included an interaction term between year and queen stock (Table S10), where colonies headed by different queen stocks did not differ in 2017, but in 2018, colonies headed by NZ queens had 154.6% more chalkbrood (e.g., Mean: 109.90 ± 24.01 brood cells showing clinical signs of chalkbrood) than colonies headed by BC (Mean: 11.56 ± 1.24) or HI (Mean: 14.08 ± 6.24) queens regardless of apiary site.

By the end of the second production year (i.e., August 2018), one third of the colonies (N = 44) remained in the project (Fig. S3). While queen stock was not included in the top model for predicting queen introductory success, apiary site was (Table S11). The proportion of successfully introduced queens was lower at the South RS apiary (Mean ± SE: 56.76% ± 8.26) compared to the North (Mean ± SE: 85.00% ± 4.65) apiary but did not differ between North and South BK (Mean ± SE: 78.12% ± 7.42) apiaries or South RS and South BK apiaries (Table S11).

**Figure 3:**
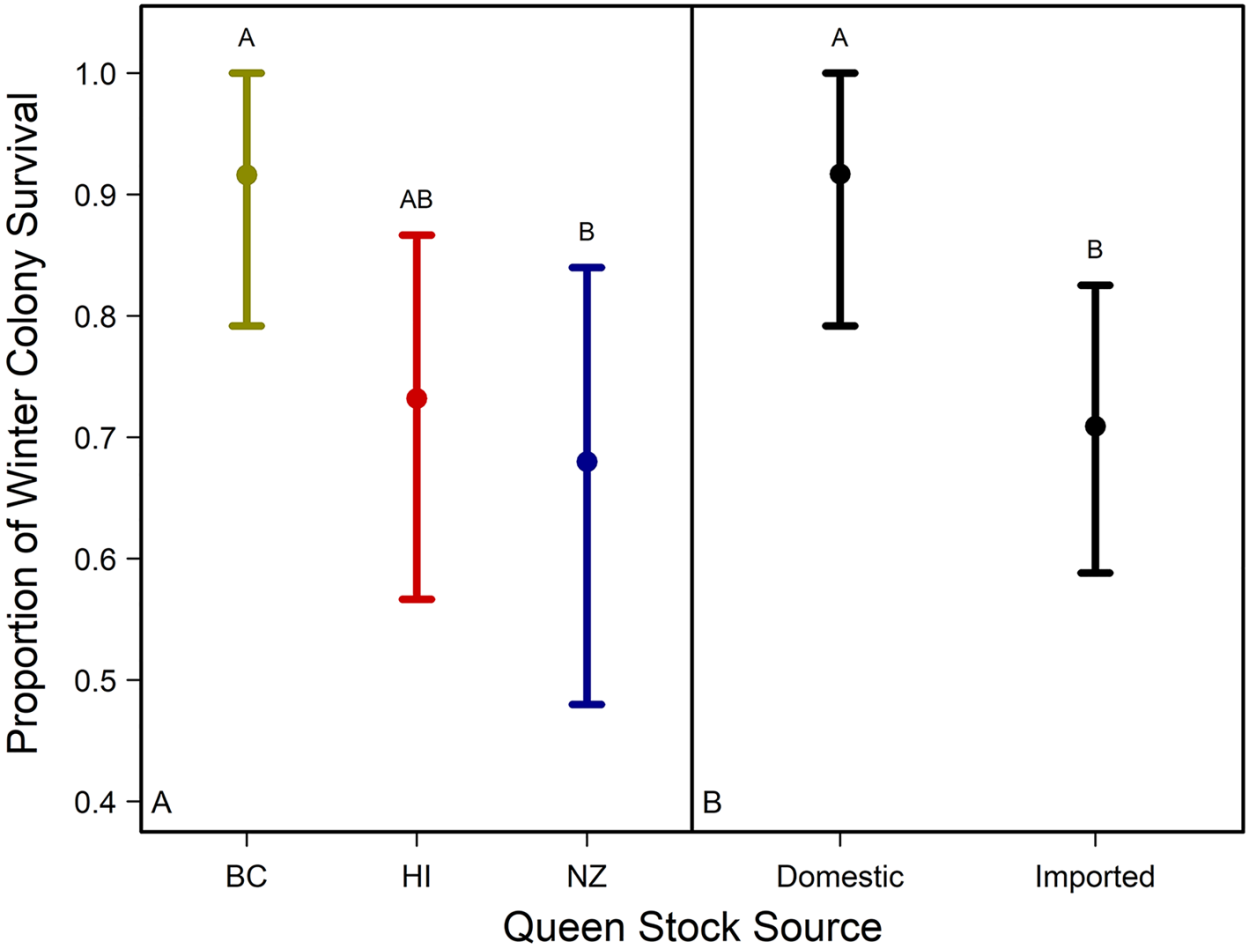
Mean and 95% confidence intervals of 20,000 nonparametric bootstrapped estimates of honey bee winter colony survival for colonies headed by different queen stocks (**A**) (e.g., BC, HI and NZ) (Fig. S1) and (**B**) (e.g., domestic (i.e., BC queens) and imported (i.e., pooling HI and NZ queens). Different letters indicate significant variation in mean winter colony survival among queen stocks (*N* = 79) (Fig. S4).

Winter mortality was not different across sites, but queen stock was included in the top model for winter colony losses (Table S12). Colonies headed by BC, HI, and NZ queens had 91.7 ± 0.05%, 73.3 ± 0.08%, and 68.0 ± 0.10% mean ± SE winter colony survival, respectively. After performing a nonparametric bootstrap on winter colony survival (Fig. S1), BC queens had 29% higher winter colony survival than colonies headed by NZ queens (Fig. 3a). Mean winter colony survival did not differ among colonies headed by HI or NZ queens or among colonies headed by BC or HI queens (Fig. 3a), however, after pooling the data by ‘domestic’ (i.e., BC) and ‘imported’ (i.e., HI and NZ) queen stocks and performing another nonparametric bootstrap (Fig. S4), we found colonies headed by domestic queens were 25% more likely to survive winter than colonies headed by imported queens (Table S13, Fig. 3b).

**Figure 4:**
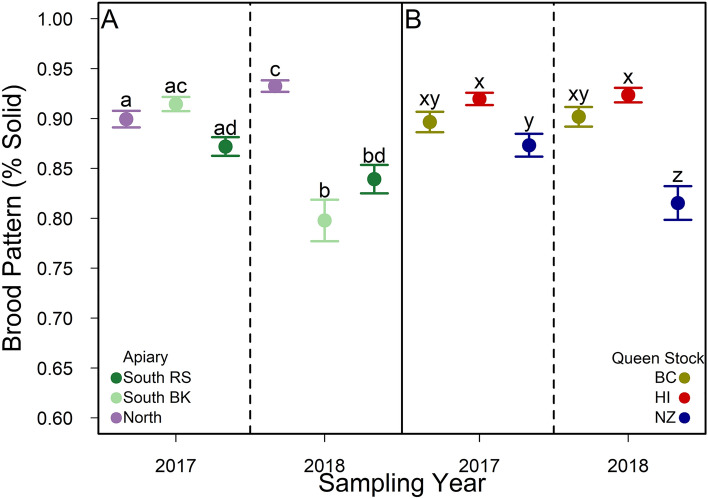
(**A**) Observed mean (± SE) solid brood pattern scores assayed in 2017 and 2018 from colonies located at three experimental apiary sites (i.e., North, South BK, and South RS in purple, light green, and dark green, respectively) in Alberta, Canada. Different letters among apiary sites across sampling years indicate significant differences *p* < 0.05. p values were adjusted for Type I error using the Bonferroni method (Table S14). (**B**) Observed mean (± SE) solid brood pattern scores assayed in 2017 and 2018 from colonies headed by three queen stocks (e.g., British Columbia, Hawaii, and New Zealand in yellow, red, and blue, respectively). Different letters among queen stocks across sampling years indicate significant differences *p* < 0.05. p values were adjusted for Type I error using the Bonferroni method (Table S14).

Solid brood pattern scores were more variable in 2018 compared to 2017, and differences in brood pattern scores among apiary sites and queen stocks depended on year (Table S14, Fig. 4a,b, respectively). Specifically, we found small differences in solid brood pattern scores among queen stocks and among sites in 2017, with slightly higher solid brood pattern scores in colonies headed by BC and HI queens compared to colonies headed by NZ queens (Fig. 4b). In 2018, we found larger differences in solid brood pattern scores among apiary sites and among colonies headed by different queen stocks. Specifically, north colonies had 10–17% higher solid brood pattern scores than colonies located at the two south apiaries and colonies headed by BC or HI queens had 12–15% higher solid brood pattern scores than colonies headed by NZ queens (Fig. 4b).

**Figure 5:**
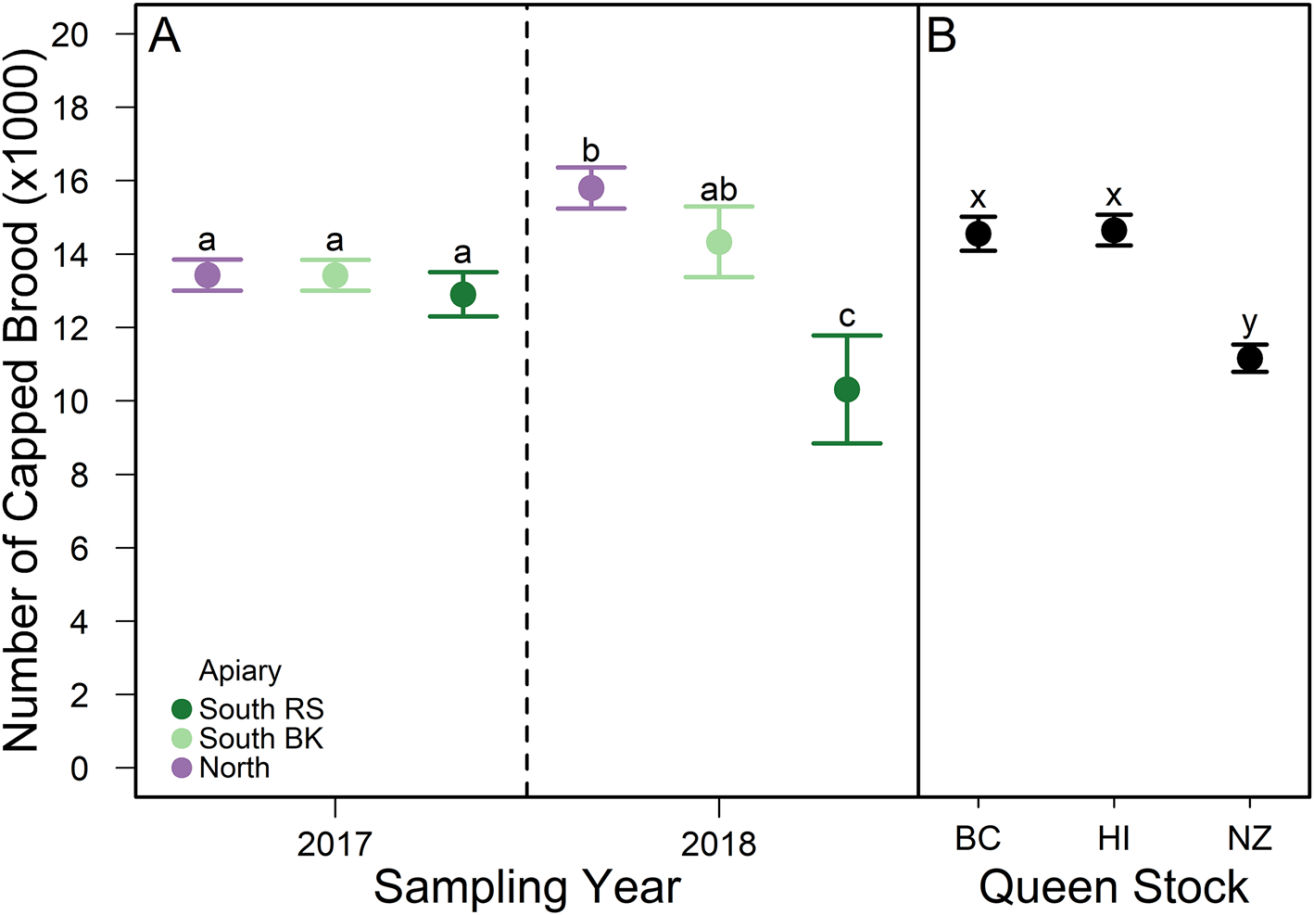
(**A**) Observed mean ± SE number of capped brood estimated from HoneyBeeComplete software for colonies located at three experimental sites (i.e., North, South BK, and South RS in purple, light green, and dark green, respectively) in Alberta, Canada in 2017 and 2018. Different letters among apiary sites across sampling years indicate significant differences *p* < 0.05 (Table S16). *p* values were adjusted for Type I error using the Bonferroni method. (**B**) Observed mean ± SE number of capped brood estimated for colonies of three queen stocks (i.e., BC, HI, and NZ). Different letters among queen stocks indicate significant differences, *p* < 0.05 (Table S16). p values were adjusted for Type I error using the Bonferroni method.

**Figure 6:**
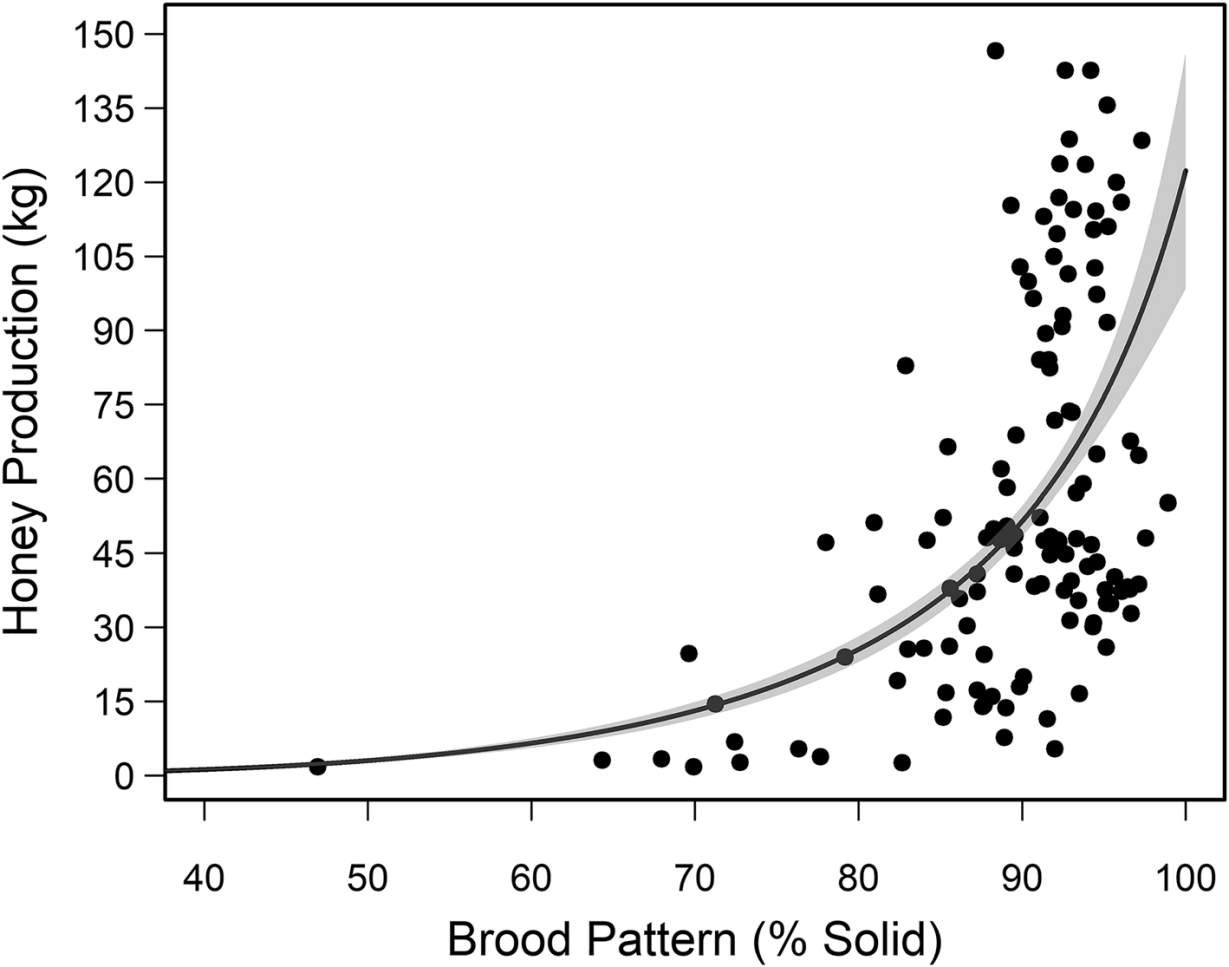
Observed mean honey production (kg) and solid brood pattern score (%) (points) with model fitting ± SE (line) characterized by a Gamma inverse link error distribution (Table S15). Experimental apiaries were set up in Beaverlodge, Alberta (North) and Lethbridge, Alberta (South BK and South RS). Data from 81 and 44 colonies from 2017 and 2018, respectively, across all sites and queen stocks.

Brood pattern solidness was positively correlated with the number of capped brood cells and adult bees estimated from photographs, and with cluster score (Table S15). We found similar trends across queen stocks, sites, and years for each of these colony performance parameters. The number of capped brood cells per colony did not vary by site in 2017 but did in 2018 with more capped brood cells in North and South BK colonies than South RS colonies (Fig. 5a and Table S16). BC and HI queen-led colonies had significantly more capped brood cells than NZ queen-led colonies (Fig. 5b). Across all apiary sites, queen stocks, and sampling years, brood pattern solidness shows a positive nonlinear correlation with honey production, where brood pattern scores below 85% correlate to lower honey production (Mean: 27.13 ± 6.02 kg), and scores 85% or higher correlate to large variation in honey production, but on average higher production (Mean: 61.92 ± 3.63kg) of honey (Table S15, Fig. 6).

**Figure 7:**
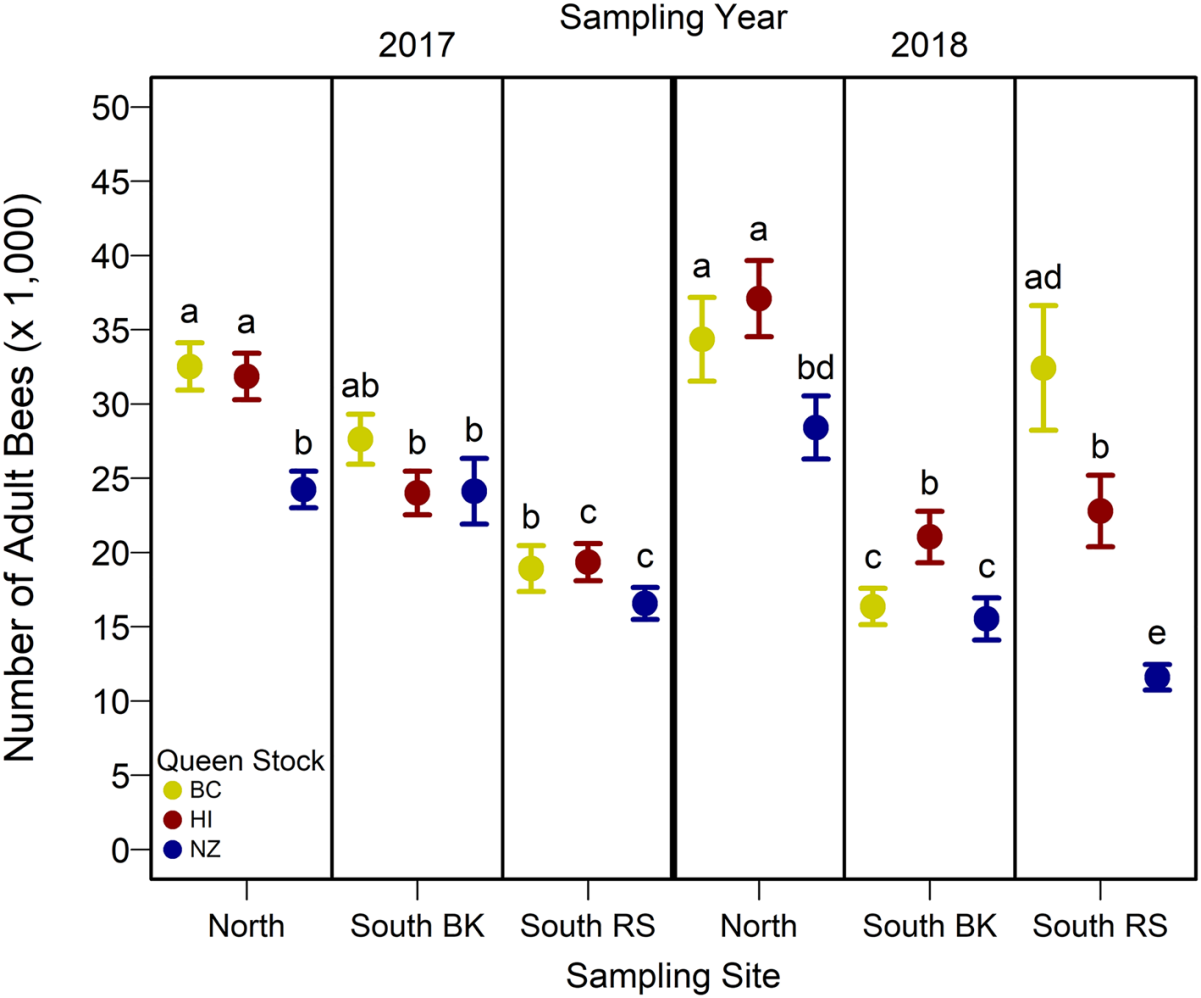
Observed mean (± SE) number of adult bees estimated from colonies of three queen stocks (i.e., BC, HI, and NZ) located at three experimental apiary sites (i.e., North, South BK, and South RS) in Alberta, Canada in 2017 and 2018 using a photo ladder^17^. Different letters among queen stocks across apiary site indicate significant differences *p* < 0.05 (Table S17). *p* values were adjusted for Type I error using the Bonferroni method.

Adult bee populations were predicted by an interaction between apiary site, queen stock, and sampling year (Table S17). In 2017, adult bee populations differed more among apiary site than queen stock, although colonies headed by NZ queens had 20–45% lower adult bee populations than colonies headed by either BC or HI queens at the North and South RS sites, respectively (Fig. 7). In 2018, adult bee populations differed more among queen stock than apiary site, although, colonies in the north were generally larger than colonies at the two south apiaries (Fig. 7). In 2018, colonies headed by NZ queens had smaller adult bee populations in the North and South RS apiary sites compared to colonies headed by BC and HI queens at these sites (Fig. 7), however, at the South BK apiary, colonies headed by NZ queens were smaller than colonies headed by HI queens but did not differ from colonies headed by BC queens (Fig. 7).

Cluster sizes were predicted by an interaction between queen stock and apiary site and an interaction term between site and year, where 2017 were the fall cluster scores and 2018 were the spring cluster scores (Fig. S5 and Table S18). Fall cluster sizes were more variable across sites (Fig. S5a and Table S18), with South BK colonies having the highest cluster scores (Mean: 8.7 ± 0.29), compared to North (Mean: 4.10 ± 0.20) and South RS (Mean: 5.20 ± 0.51) colonies which had similar cluster scores. Spring cluster scores (Mean: 4.76 ± 0.22) were not different across sites (Fig. S5a). Cluster sizes were similar among queen stocks at the North and South BK sites; however, the cluster size was 46–58% smaller for colonies headed by NZ queens compared to colonies headed by HI and BC queens, respectively at South RS sites (Fig. S5b).

Honey production was affected by an interaction between site and year (Table S19). In 2017, honey production was highly variable among the three apiary sites. North colonies (100.25 ± 3.9 kg) produced 88–142% more honey than South BK (39.02 ± 1.46 kg) and South RS colonies (16.98 ± 2.83 kg), respectively (Table S20). In 2018, North colonies (50.41 ± 3.57 kg) produced 55–62% more honey than South RS (28.62 ± 4.95 kg) and South BK (26.66 ± 5.02 kg) colonies, respectively, however, the two south sites did not differ in their honey production (Table S20).

## Discussion

Over a two-year field study, domestic and imported queen stocks were evaluated at two locations: one site in Northern Alberta (Beaverlodge) and two sites in Southern Alberta (Lethbridge). Our results suggest apiary site is a stronger predictor of colony health and productivity than queen stock in year one, but in year two, queen stock is a stronger predictor than apiary site. Most strikingly, our results show colonies headed by domestic queen stocks are 25% more likely to survive winter than colonies headed by imported queens (Fig. 3b), and colonies headed by queens from New Zealand had smaller populations, higher frequency of clinical symptoms of chalkbrood, and less solid brood patterns than colonies headed by Hawaiian and British Columbian queens.

Despite variation among queen morphological trait parameters clustering by queen stock in our principal components analysis (Fig. 1), queen morphology and fertility did not vary greatly among queen stocks overall (Table S1). In general, HI and BC queens were morphometrically comparable; however, BC queens had a wider thorax and head, and a larger spermatheca compared to HI queens. Despite HI queens having smaller spermathecae, sperm count did not differ among queen stocks and the higher sperm viability found in NZ compared to HI queens may simply be related to the natural variation in the availability of drones and drone maturity seasonally. For example, Pettis et al*.*^27^ found sperm viability in a queen’s spermatheca varies not only from queen breeder to queen breeder, but even within queen breeders seasonally (e.g., July vs September). Thus, the differences in sperm viability among our three queen stocks may simply be an artifact of natural variation in drone availability and maturity.

Other factors that could impact sperm viability in queens include temperatures experienced during queen transit^28,29^, lower drone quality, exposure to pesticides^30–32^, and queen immunity^33^. Pettis et al.^27^ found that even relatively short exposures to extreme temperatures (4 °C or 40 °C) can be detrimental to stored sperm.

Interestingly, data loggers placed in our queen shipments revealed that the two shipments of NZ queens destined for Beaverlodge and Lethbridge, Alberta, maintained an average temperature of 18.5 °C and 17.1 °C, respectively. After 8–10 h of cooling to 4.4 °C and 5 °C for Beaverlodge and Lethbridge destinations, respectively (i.e., to prepare them for temperature fluctuations during takeoff), NZ queens did not experience extreme temperatures (e.g., below 5 °C or above 40 °C). Despite this cooling period, the NZ queens arrived with high sperm viability, suggesting they were protected thermally by the worker bees, or it is possible the speed of heating and cooling also matters to sperm viability. Hawaii queen stocks experienced temperatures between 16 °C and 30 °C with an average of 23.6 °C but did not experience any periods of extreme cooling or warming. We cannot discard, however, that any temperature fluctuations experienced by these queens may have affect their performance, even without a decrease in sperm viability.

The viability of sperm stored in the spermatheca declines as queens age^9,34^ and therefore, finding a positive correlation among sperm count and viability at the ‘end’ of a queen’s life and not for newly mated queens, is not surprising. However, the positive correlation between sperm count and sperm viability among queens at the end of the experiment should be interpreted carefully because of small sample sizes and sampling bias (i.e., only surviving queens were analyzed). Lower versus higher sperm viability in queens from colonies identified as “failing” or “healthy”, respectively, have been reported^27,33^, however, two queens identified as failing two months prior to the end of the experiment were sampled and had sperm counts of 6.6 and 8.2 million with 89.3% and 90.2% sperm viability, respectively, suggesting the queens were failing for reasons other than sperm count or sperm viability.

Arguably, the most prominent difference among the queens from the different stocks was the smaller body and ovary weights of the NZ queens compared to HI and BC queens (Table S1). Queen body size is an important indicator of queen health and has been correlated with queen attractiveness to worker bees, acceptance into a new unrelated colony, and reproductive success (reviewed in^5^); The reduced size of NZ queens compared to HI and BC queens is likely a result of spending a longer period caged prior to and during transit. Queens are physiologically capable of inactivating their ovaries and partially resorbing their eggs (^35^ as referenced in^36^) and do so when caged outside their colony^37^. NZ queens destined for Canada are often banked and in transit longer than BC or HI queens. NZ, HI, and BC queens can spend between 4–5, 1–3, and 1–2 days in transit, respectively, and therefore the inability to actively lay eggs, interact with their environment, and be fed the nutrients (e.g., fats and proteins) required for oogenesis, may have caused NZ queens to inactivate their ovaries resulting in smaller ovaries and overall body weight. Importantly, McAfee et al*.*^33^ showed that after two weeks of caging, when released inside a colony, imported queens regain their ovary masses, suggesting smaller ovaries are not a quality trait of imported queens.

While many of the assays we performed showed apiary site and/or year interactions, queen stock source was a predictor of winter survival (Fig. 3), solid brood pattern (Fig. 4), the number of capped brood cells (Fig. 5) adult bee populations (Fig. 7), and the abundance of chalkbrood disease (Table S10). Colonies headed by domestic queens (i.e., BC queens) were more likely to survive winter in Alberta than colonies headed by imported queens (e.g., HI and NZ). Similar *Varroa* levels across apiary sites and queen stocks throughout the experiment suggest that *Varroa* tolerance or resistance is not the cause of this effect. Reliance on stocks selected for and bred in locations without harsh winters are likely contributing to the high winter colony mortality experienced by Alberta beekeepers, for example, European studies have found locally bred queens survive longer^38^ with lower overwintering mortality^39^ than non-local queens. Further, after importing honey bee stocks from various climatic origins (e.g., USA (i.e., California and Hawaii), Chile, New Zealand, and Canada (i.e., Saskatchewan and Ontario)) to northern Alberta, a Canadian study found that the expression of energy-related mitochondrial pathways differed among stock populations that had originated from colder and warmer climates^40^, suggesting some metabolic pathways in bees may not locally adapt to new climactic regions when moved from contra-season breeding origins. Increasing support to domestic queen breeders to overcome the challenges of breeding domestic queens earlier in spring is an important avenue of future research^41^.

We found that the variation among colony health and productivity parameters measured clustered by site (Fig. 2), where colonies at the North location differed from colonies at the two South sites. In general, we found adult bee populations and honey yields were larger at the North site. While in the same Canadian province, the North and South locations experience vastly different beekeeping conditions. Beaverlodge is 760 km (472 miles) northwest of Lethbridge and experiences harsher winters, with nearly twice as many days where daily maximum temperatures are below freezing (e.g., 86 days vs. 48.1 days^42^). However, while Lethbridge experiences milder winters and warmer, longer summers, this has not historically translated into better summer beekeeping conditions. Instead, the abundant forage in the North combined with longer daylight hours, (e.g., 17.22h and 16.16h in Beaverlodge and Lethbridge, respectively, at the summer solstice^42^) has historically resulted in larger colonies that produce more honey. Szabo and Lefkovich^43^ found 4.5 times more honey was produced in Northern Alberta than in Southern Alberta (e.g., 149.8 kg in Falher, Alberta vs. 32.6 kg in Scandia, Alberta), and found larger populations (e.g., 11.9 frames of bees vs. 6.6 frames of bees). Similarly, the Alberta Beekeepers’ Survey^7^ reports that the Peace Region (North) has outproduced (yield per colony) the South Region (Lethbridge) for each of the last eight years, on average producing double the amount of honey.

Seasonal and annual differences in weather and nectar and pollen flows among the North and South locations are likely behind the site effects we found. For example, the higher hygienic behavior scores observed in the North in 2018 are likely due to the assays being conducted later in June, when North colonies were experiencing nectar flow conditions. Nectar flows can cause increased nest cleaning and thus, hygienic behaviour^14^. Hygienic behavior has a strong genetic basis^44^, and it is interesting that we found no differences queen stocks from widely different areas in either year of the trial. Site effects on queen stock performance are also consistent with anecdotal reports from Albertan beekeepers that colonies of different queen stocks perform differently depending on the location of the apiary, where some colonies are more defensive in some areas of Alberta than others and at different times throughout the season. Limited research from other countries suggests honey bee defensive behaviour is negatively correlated with altitude^45,46^ and differs across latitude^47^. However, given defensive behaviour did not differ strictly among North and South locations, other environmental factors are likely responsible.

The NZ stock performed poorly in our colony health and productivity assessments compared to colonies headed by other queen stocks. The NZ queen-led colonies had significantly less solid brood patterns than BC and HI queen stocks, despite NZ queens having higher sperm viability and similar sperm counts. Solid brood patterns are typically associated with strong colonies and our positive correlations between solid brood pattern and the number of capped brood cells and adult bees support this (Table S15). Poor brood patterns can be attributed to many causes including, poor egg laying by the queen (i.e., she does not lay eggs in every cell or the eggs she lays are not viable or healthy), hygienic behaviour of the workers that remove dead or diseased larvae, insufficient food, or cannibalism of diploid drones^48^. However, brood pattern evaluation is a metric commonly employed by beekeepers to evaluate queen quality. Importantly, Lee et al.^49^ conducted a reciprocal transplant experiment of queens producing poor- and high-quality brood patterns in colonies and found that queens with good brood patterns initially produced poor brood patterns when they were introduced to colonies that had previously produced poor brood patterns. Thus, once queens are introduced and laying in colonies, poor brood pattern is not a reliable indicator of a poor laying queen (although it can be).

Finding the cause of a poor brood pattern is important and necessary to remediate the problem because brood patterns scores below 85% solid can significantly predict lower honey production (Fig. 6). It is also critical that beekeepers carefully examine brood frames and bottom board for signs of brood disease. The strong negative correlation between the abundance of brood cells showing clinical signs of chalkbrood disease and solid brood pattern score for our South colonies (Table S15) suggests our low solid brood pattern scores (at least at the South apiary sites) was likely due to disease susceptibility not queen quality / fertility. The higher chalkbrood levels for colonies at the South sites headed by NZ queens may be related to differences in immunity to chalkbrood variants in Southern Alberta that may or may not be present in New Zealand. For example, Gerdts et al*.*^50^ noted a single haplotype of chalkbrood (*Ascosphaera apis)* (i.e., strain A) in New Zealand, but noted two haplotypes of chalkbrood (i.e., strain A and strain B) in North America. Immunity to chalkbrood has been shown to depend on both honey bee genotype and *A. apis* haplotypes^51^. While we did not genotype our bees or chalkbrood, a case could be made for differences in immunity to chalkbrood among queen stocks given the significant differences in chalkbrood presence among colonies headed by different queen stocks and recent accounts of chalkbrood variants around the world^50^. Given the number of South colonies plagued by chalkbrood in 2018, susceptibility to chalkbrood likely had important colony-level impacts, where colonies headed by NZ queens had significantly lower solid brood patterns, fewer capped brood cells per colony, and fewer adult bees than colonies headed by BC or HI queen stocks. It is important for beekeepers to recognize that chalkbrood can have strong impacts on colony productivity and survival.

In conclusion, measuring sperm viability and count of queens at shipment can be useful to identify problems, such as those caused by poor mating conditions or temperature stress during shipment, but high sperm count and viability are not reliable indicators that queens will perform well, especially over multiple production seasons. Queen measurements are variable even within a single shipment, and good average measurements do not preclude poor-performing outliers. It is also important to consider that sampling a subset of queens in a queen shipment gives only a “snapshot” picture of the measures of those queens at that time. Very large queen shipments will include queens from many mating yards or dates and may therefore be more variable. Given that the testing is expensive, labour-intensive, and time-constrained, it is important to consider how many queens would need to be sampled to give an accurate estimate of the shipment mean, and the value of the information gained.

Lastly, regardless of queen stock or site, two-thirds of queens tested from commercial imported and domestic stocks did not survive two production seasons in Alberta, Canada. We found domestic queen stocks were 25% more likely to survive winter in Alberta than imported queen stocks. We also found the NZ queen stock performed poorly overall, with most colonies at the South apiary sites suffering from high chalkbrood loads that translated into poor brood patterns, and low bee populations. Although limited in the number of stocks and queens, this is the first report of a detailed comparison of local and imported stocks in Western Canada. While our results confirm the general observation that local stocks overwinter better than imported stocks in Canada, larger studies using additional stocks are needed to generalize our imported vs domestic queen results. Moving forward, it is important to consider the possible mismatches in disease immunity and climate conditioning of imported queen stocks heading colonies in Alberta and work towards domestic stock self sufficiency.

### Supplementary Information

Supplementary Information.

## Data Availability

The data used for this study is available from the corresponding author on reasonable request.
