# Supplementary material for: Queen quality, performance, and winter survival of imported and domestic honey bee queen stocks
Source: Sci Rep. 2023 Oct 12;13:17273. doi: 10.1038/s41598-023-44298-x (PMC10570385; doi:10.1038/s41598-023-44298-x)
Supplement: Supplementary file 1 — Supplementary Information. [file 41598_2023_44298_MOESM1_ESM.pdf]

Supplementary Information

Queen quality, performance, and winter survival of imported and domestic honey bee queen stocks

Holmes, L.A., L.P. Ovinge, J.D. Kearns, A. Ibrahim, P. Wolf Veiga, M.M. Guarna, S.F. Pernal, and S.E. Hoover

Table S1: Model selection using Akaike information criterion (AIC) for the effect of queen stock (Stk) (e.g., British Columbia (BC), Hawaii (HI), and New Zealand (NZ)) on various queen morphological traits measured for honey bee queens, (Y) at the start of our experiment.

Model	ΔAIC or ΔqAIC	Df	Weight	Residual Deviance	No. of Observations	Null Deviance
**Weight**						
Y ~ Stk	0.0	4	1	0.20	45	0.35
Y ~ 1	19.68	2	0	0.35		
Error Distribution: Gamma with inverse link						
**Head Width**						
Y ~ Stk	0.00	4	0.96	0.79	45	1.02
Y ~ 1	6.64	2	0.04	1.02		
Error Distribution: gaussian						
**Thorax Length**						
Y ~ Stk	0.00	4	1	1.72	45	2.13
Y ~ 1	230.3	2	< 0.001	2.13		
Error Distribution: quasi						
**Thorax Width**						
Y ~ Stk	0.00	4	1	0.78	45	1.24
Y ~ 1	1110.1	2	< 0.001	1.24		
Error Distribution: quasi						
**Spermatheca Volume**						
Y ~ Stk	0.00	4	1	0.33	45	0.57
Y ~ 1	3158.6	2	< 0.001	0.57		
Error Distribution: quasi						
**Sperm Count**						
log(Y) ~ Stk	0.00	4	0.65	2.19	44	2.51
log(Y) ~ 1	1.21	2	0.35	2.51		
**Sperm Viability**						
Y ~ Stk	0.0	3	0.80	20.53	44	23.82
Y ~ 1	2.7	1	0.20	23.82		
Error Distribution: quasi-Poisson with log link						
**No. of Ovarioles per Ovary**						
Y ~ 1	0.0	1	0.70	59.86	30	59.86
Y ~ Stk	1.7	3	0.30	55.02		
Error Distribution: quasi-Poisson with log link						
**Ovary wet weight**						
Y ~ Stk	0.00	4	0.998	309.05	30	563.91
Y ~ 1	12.89	2	0.002	563.91		
Error Distribution: quasi						

Table S2: Model selection using Akaike information criterion (AIC) for the effect of queen stock (Stk) (e.g., BC, HI, and NZ) on the correlation between sperm viability, (Y) and sperm count (SC) fit with a gaussian error distribution and fit to 44 observations with 2007.5 null deviance.

Model	ΔAIC	Df	Weight	Residual Deviance
Y ~ Stk	0.0	4	0.518	1724.1
Y ~ 1	1.96	2	0.194	2007.5
Y ~ log(SC) + Stk	2.30	5	0.164	1714.1
Y ~ log(SC)	2.86	3	0.124	1944.0

Table S3: Model selection using Akaike information criterion (AIC) for the effect of apiary site (S) (e.g., North and South) and queen stock (Stk) (e.g., BC, HI, and NZ) on end-of-life sperm counts, (Y), fit with a negative binomial error distribution due to overdispersion after fitting a Poisson distribution and fit to 28 observations with 37.98 null deviance. General linear hypothesis post-hoc testing on the top model fit (i.e., Y ~ S) selected by Akaike Information Criterion (AIC) is included with adjusted p values for Type I error using the Bonferroni method.

**Model**	**ΔAIC**	**Df**	**Weight**	**Residual Deviance**
Y ~ S	0.0	3	0.635	29.4
Y ~ 1	1.73	2	0.268	38.0
Y ~ S + Stk	4.99	5	0.052	29.4
Y ~ Stk	5.59	4	0.039	29.6
Y ~ S * Stk	9.20	7	0.006	29.3
**Post-hoc Comparison**	**Std. Error**	**z value**	**Pr(>|z|)**	
North - South	0.21	2.13	0.033	

Table S4: Model selection using Akaike information criterion (AIC) for the effect of apiary site (S) (e.g., North, South BK, and South RS), queen stock (Stk) (e.g., BC, HI, and NZ), and sampling year (Yr) (e.g., 2017, and 2018) on hygienic behaviour, (Y), fit with a gaussian error distribution and fit to 138 observations with 128.17 null deviance. Top four models and the null model are shown.

Model	ΔqAIC	Df	Weight	Residual Deviance
sqrt(Y) ~ S * Yr	0.0	7	0.866	97.04
sqrt(Y) ~ S * Yr + Stk	3.95	9	0.120	96.63
sqrt(Y) ~ S * Yr + Stk * Yr	8.58	11	0.012	96.58
sqrt(Y) ~ S * Yr + S * Stk	12.51	13	0.002	95.95
sqrt(Y) ~ 1	27.40	5	0.000	128.17

Table S5: Model selection using quasi Akaike information criterion (qAIC) for the effect of apiary site (S) (e.g., North, South BK, and South RS), queen stock (Stk) (e.g., BC, HI, and NZ), and sampling year (Yr) (e.g., 2017, and 2018) on defensive behaviour, (Y), fit with a quasi-Poisson error distribution due to overdispersion and fit to 272 observations with 4949.6 null deviance. Top four models and the null model are shown.

Model	ΔqAICc	Df	Weight	Residual Deviance
Y ~ S * Yr	0.0	6	0.777	3291.2
Y ~ S * Yr + Stk	3.75	8	0.119	3287.6
Y ~ S + Yr	5.62	4	0.047	3431.1
Y ~ Stk * Yr + S * Yr	7.36	10	0.020	3282.0
Y ~ 1	104.07	1	0.000	4949.6

Table S6: General linear hypothesis post-hoc testing on the top model fit selected by Akaike Information Criterion (AIC) for the effect of apiary site (e.g., North, South BK, and South RS) and sampling year (e.g., 2017 and 2018) on hygienic behaviour and defensive behaviour. Adjusted p values for Type I error using the Bonferroni method are presented.

	Post-hoc Comparisons	Std. Error	z value	Pr(>|z|)
**Hygienic Behaviour: Apiary x Sampling Year Interaction**				
	South BK in 2017 – North in 2017	0.23	-0.361	0.999
	South RS in 2017 – North in 2017	0.24	1.988	0.344
	North in 2018 – North in 2017	0.22	4.147	< 0.001
	South BK in 2018 – North in 2017	0.25	0.077	1.000
	South RS in 2018 – North in 2017	0.26	-3.008	0.031
	South RS in 2017 – South BK in 2017	0.27	2.041	0.315
	North in 2018 – South BK in 2017	0.25	3.865	0.002
	South BK in 2018 – South BK in 2017	0.28	0.359	0.999
	South RS in 2018 – South BK in 2017	0.29	-2.391	0.156
	North in 2018 – South RS in 2017	0.26	1.636	0.569
	South BK in 2018 – South RS in 2017	0.29	-1.553	0.624
	South RS in 2018 – South RS in 2017	0.30	-4.211	< 0.001
	South BK in 2018 – North in 2018	0.27	-3.202	0.017
	South RS in 2018 – North in 2018	0.28	-5.978	< 0.001
	South RS in 2018 – South BK in 2018	0.31	-2.581	0.099
**Defensive Behaviour: Apiary x Sampling Year Interaction**				
	South BK in 2017 – North in 2017	2.52	0.06	1.000
	South RS in 2017 – North in 2017	2.65	0.56	0.994
	North in 2018 – North in 2017	2.38	6.17	< 0.001
	South BK in 2018 – North in 2017	2.77	4.97	< 0.001
	South RS in 2018 – North in 2017	2.83	1.20	0.833
	South RS in 2017 – South BK in 2017	3.03	0.44	0.998
	North in 2018 – South BK in 2017	2.79	5.22	< 0.001
	South BK in 2018 – South BK in 2017	3.13	4.35	< 0.001
	South RS in 2018 – South BK in 2017	3.19	1.02	0.908
	North in 2018 – South RS in 2017	2.91	4.54	< 0.001
	South BK in 2018 – South RS in 2017	3.24	3.79	0.002
	South RS in 2018 – South RS in 2017	3.29	0.59	0.992
	South BK in 2018 – North in 2018	3.02	-0.31	0.999
	South RS in 2018 – North in 2018	3.08	-3.67	0.003
	South RS in 2018 – South BK in 2018	3.39	-3.05	0.027

Table S7: Model selection using Akaike information criterion (AIC) for the effect of apiary site (S) (e.g., North, South BK, and South RS), queen stock (Stk) (e.g., BC, HI, and NZ), and sampling year (Yr) (e.g., 2017, and 2018) on *Varroa destructor* mite counts, (Y), fit with a gaussian error distribution and fit to 170 observations with 71.36 null deviance. Top four models and the null model are shown. General linear hypothesis post-hoc testing on the top model (i.e., Y ~ Yr) fit selected by Akaike Information Criterion (AIC) for the effect of sampling year (e.g., 2017 and 2018) on *Varroa destructor* mite counts with adjusted p values for Type I error using the Bonferroni method.

Model	ΔAIC	Df	Weight	Residual Deviance
Y ~ Yr	0.00	3	0.648	57.65
Y ~ S + Yr	2.80	5	0.160	57.17
Y ~ Stk + Yr	3.97	5	0.089	57.57
Y ~ S * Yr	4.91	7	0.056	56.43
Y ~ 1	34.18	2	0.000	71.36
**Post-hoc Comparison**	**Std. Error**		**z value**	**Pr(>|z|)**
2017 – 2018	0.09		-6.32	< 0.001

Table S8: Model selection using Akaike information criterion (AIC) for the effect of apiary site (S) (e.g., North, South BK, and South RS), queen stock (Stk) (e.g., BC, HI, and NZ), and sampling year (Yr) (e.g., 2017, and 2018) on *Vairimorpha sp. (Nosema)* spore counts, (Y), fit with a gaussian error distribution and fit to 170 observations with 3747.7 null deviance. Top four models and the null model are shown.

Model	ΔAIC	Df	Weight	Residual Deviance
log(Y) ~ S * Yr + S * Stk	0.00	13	0.702	1730.4
log(Y) ~ Stk * Yr + S * Yr + S * Stk	3.54	15	0.120	1717.8
log(Y) ~ S * Yr + Stk	4.29	9	0.082	1873.5
log(Y) ~ S * Yr	5.73	7	0.040	1939.2
log(Y) ~ 1	107.11	2	0.000	3747.7

Table S9: Model selection using Akaike information criterion (AIC) for the effect of apiary site (S) (e.g., South BK and South RS) and sampling year (Yr) (e.g., 2017, and 2018) on the proportion of colonies showing clinical signs of chalkbrood disease, (Y), fit with a binomial error distribution with a logit link function and fit to 78 observations with 102.95 null deviance. Top four models and the null model are shown.

Model	ΔAIC	Df	Weight	Residual Deviance
Y ~ S + Yr	0.00	3	0.406	71.07
Y ~ S * Yr	1.79	4	0.166	70.64
Y ~ S + Yr + Stk	2.05	5	0.145	68.62
Y ~ S	3.25	2	0.080	76.49
Y ~ 1	27.60	1	0.000	102.95

Table S10: Model selection using Akaike information criterion (AIC) for the effect of apiary site (S) (e.g., South BK and South RS) and queen stock (e.g., BC, HI, and NZ) on the number of brood cells showing clinical signs of chalkbrood disease, (Y), fit with a gaussian error distribution and fit to 29 observations with 58.24 null deviance. Top four models and the null model are shown. The table also shows the general linear hypothesis post-hoc testing on the top model fit selected by Akaike Information Criterion (AIC) for the effect of queen stock (e.g., BC, HI, and NZ) and sampling year (e.g., 2017 and 2018) on the log of the mean number of brood cells showing clinical signs of chalkbrood disease with adjusted p values for Type I error using the Bonferroni method.

Model	ΔAIC	Df	Weight	Residual Deviance
log(Y) ~ Stk * Yr	0.00	7	0.470	22.03
log(Y) ~ Stk * Yr + S * Yr	1.97	9	0.176	17.81
log(Y) ~ Stk * Yr + S	2.61	8	0.128	21.10
log(Y) ~ Stk + Yr	3.43	5	0.085	31.27
Y ~ 1	13.32	2	0.001	58.24
**Post-hoc Comparisons**	**Std Error**		**z value**	**Pr(>|z|)**
HI queens in 2017 – BC queens in 2017	0.75		-1.15	0.857
NZ queens in 2017 – BC queens in 2017	0.63		-0.87	0.953
BC queens in 2018 – BC queens in 2017	0.75		0.31	1.000
HI queens in 2018 – BC queens in 2017	0.63		-0.17	1.000
NZ queens in 2018 – BC queens in 2017	0.61		3.66	0.003
NZ queens in 2017 – HI queens in 2017	0.69		0.45	0.998
BC queens in 2018 – HI queens in 2017	0.80		1.37	0.741
HI queens in 2018 – HI queens in 2017	0.69		1.09	0.885
NZ queens in 2018 – HI queens in 2017	0.68		4.60	< 0.001
BC queens in 2018 – NZ queens in 2017	0.69		1.13	0.866
HI queens in 2018 – NZ queens in 2017	0.57		0.78	0.971
NZ queens in 2018 – NZ queens in 2017	0.54		5.13	< 0.001
HI queens in 2018 – BC queens in 2018	0.69		-0.50	0.996
NZ queens in 2018 – BC queens in 2018	0.68		2.98	0.034
NZ queens in 2018 – HI queens in 2018	0.54		4.33	< 0.001

Table S11: Model selection using Akaike information criterion (AIC) for the effect of apiary site (S) (e.g., North, South BK, and South RS) and queen stock (Stk) (e.g., BC, HI, and NZ) on queen introductory success, (Y), fit with a binomial error distribution and logit link function and fit to 129 observations with 144.53 null deviance. Queen introductory success was assessed after a 42-day period (i.e., the length of two brood cycles). Top four models and the null model are shown. The table also shows the general linear hypothesis post-hoc testing on the top model fit selected by Akaike Information Criterion (AIC) for the effect of apiary site on queen introductory success with adjusted p values for Type I error using the Bonferroni method.

Model	ΔAIC	Df	Weight	Residual Deviance
Y ~ S	0.00	3	0.823	134.96
Y ~ S + Stk	4.16	5	0.103	134.83
Y ~ 1	5.41	1	0.055	144.53
Y ~ S * Stk	8.54	9	0.012	130.18
Y ~ Stk	9.49	3	0.007	144.45
**Post-hoc Comparisons**	**Std. Error**		**z value**	**Pr(>|z|)**
South BK – North	0.56		-0.82	1.000
South RS – North	0.49		-2.98	0.009
South RS – South BK	0.54		-1.85	0.193

Table S12: Model selection using Akaike information criterion (AIC) for the effect of apiary site (S) (e.g., North, South BK, and South RS) and queen stock (Stk) (e.g., BC, HI, and NZ) on winter colony loss, (Y), fit with a binomial error distribution and logit link function and fit to 79 observations with 84.79 null deviance. Colonies that did not survive or maintain at least four frames of bees by May 21, 2018 [1] were pulled from the experiment. Top four models and the null model are shown.

Model	ΔAIC	Df	Weight	Residual Deviance
Y ~ Stk	0.00	3	0.471	79.91
Y ~ 1	0.62	1	0.346	84.79
Y ~ S + Stk	3.16	5	0.097	78.56
Y ~ S	3.82	3	0.070	83.72
Y ~ S * Stk	6.83	9	0.016	72.44

Table S13: Model selection using Akaike information criterion (AIC) for the effect of apiary site (S) (e.g., North, South BK, and South RS) and queen stock (Stk) (e.g., domestic (BC) and imported (HI and NZ pooled)) on winter colony loss, (Y), fit with a binomial error distribution and a logit link function and fit to 79 observations with 84.79 null deviance. Colonies that did not survive or maintain at least four frames of bees by May 21, 2018 [1] were pulled from the experiment. Top four models and the null model are shown.

Model	ΔAIC	Df	Weight	Residual Deviance
Y ~ Stk	0.00	2	0.633	80.09
Y ~ 1	2.59	1	0.173	84.79
Y ~ S + Stk	3.01	4	0.141	78.12
Y ~ S	5.79	3	0.035	83.72
Y ~ S * Stk	7.08	6	0.018	78.17

Table S14: Model selection using quasi Akaike information criterion (qAIC) for the effect of apiary site (S) (e.g., North, South BK, and South RS), queen stock (Stk) (e.g., BC, HI, and NZ), and sampling year (Yr) (e.g., 2017, and 2018) on solid brood pattern score, (Y) fit with a quasipoisson error distribution and log link function due to overdispersion and fit to 583 observations with 4770.4 null deviance. Top four models are compared to the null model.

Model	ΔqAIC	Df	Weight	Residual Deviance
Y ~ Stk * Yr + S * Yr	0.00	10	0.355	3484.0
Y ~ Stk * Yr + S * Yr + S * Stk	3.35	14	0.352	3446.8
Y ~ Stk * Yr * S	4.96	18	0.240	3398.8
Y ~ S * Yr + S * Stk	5.34	18	0.034	3497.2
Y ~ 1	142.97	1	0.000	4770.4

Table S15: Model selection using Akaike information criterion (AIC) for the effect of queen stock (Stk) (e.g., BC, HI, and NZ) in a generalized linear regression between mean solid brood pattern score (SBP) and the number of capped brood (NCB), between SBP and the number of adult bees (NAB), between SBP and cluster score (CS), between SBP and the abundance of chalkbrood (CB), between CB and hygienic behaviour (HB), between SBP and HB, and between SBP and HY. Correlations including chalkbrood excluded data from the North apiary site due to no available data. The correlation between SBP and HY was well characterized by an under-dispersed Gamma with an inverse link error distribution.

**Model**	**ΔqAIC**	**df**	**Weight**	**Residual Deviance**	**R^2**
NCB ~ SBP + Stk	0.00	4	1	66651	NCB ~ SBP
NCB ~ SBP	17.02	2	0	76596	$R^2 = 0.54$
NCB ~ Stk	31.06	3	0.000	82292	$p < 0.05$
NCB ~ 1	81.02	1	0.000	107817	

NAB ~ SBP + Stk	0.00	4	0.749	172538	NAB ~ SBP
NAB ~ SBP	2.18	2	0.251	181490	$R^2 = 0.64$
NAB ~ Stk	79.97	3	0.000	291202	$p < 0.05$
NAB ~ 1	112.16	1	0.000	343589	
SBP ~ 1	0.00	1	0.621	8098.2	SBP ~ HB
SBP ~ HB	1.98	2	0.231	8043.8	$R^2 = 0.08$
SBP ~ Stk	3.50	3	0.108	6753.6	$p = 0.34$
SBP ~ HB + Stk	5.46	4	0.040	6675.1	

**Model**	**ΔAIC**	**df**	**Weight**	**Residual Deviance**	**R^2**
CS ~ SBP + Stk	0.00	5	0.643	583.05	CS ~ SBP
CS ~ Stk	2.55	4	0.179	603.44	$R^2 = 0.28$
CS ~ SBP	2.59	3	0.176	613.04	$p < 0.05$
CS ~ 1	11.81	2	0.002	665.78	
CB ~ SBP	0.00	3	0.924	41.06	CB ~ SBP
CB ~ SBP + Stk	5.01	5	0.075	40.17	$R^2 = -0.80$
CB ~ 1	15.73	2	0.000	76.98	$p < 0.05$
CB ~ Stk	15.91	4	0.000	64.74	
CB ~ Stk	0.00	4	0.486	58.00	CB ~ HB
CB ~ 1	0.92	2	0.307	73.07	$R^2 = -0.08$
CB ~ HB + Stk	2.92	5	0.113	57.75	$p = 0.68$
CB ~ HB	3.28	3	0.094	72.57	

**Model**	**ΔAIC**	**df**	**Weight**	**Residual Deviance**
HY ~ $I(SBP^{-1})^4$	0.00	3	0.755	62.65
HY ~ $I(SBP^{-1})^3$	2.72	3	0.194	59.80
HY ~ $I(SBP^{-1})^2$	5.81	3	0.041	58.37
HY ~ $I(SBP^{-1})$	9.03	3	0.008	57.02
HY ~ SBP	15.22	3	0.000	55.86
HY ~ 1	50.83	2	0.000	82.84

Table S16: Model selection using Akaike information criterion (AIC) for the effect of apiary site (S) (e.g., North, South BK, and South RS), queen stock (Stk) (e.g., BC, HI, and NZ), and sampling year (Yr) (e.g, 2017, and 2018) on mean number of capped brood, (Y), fit with a negative binomial error distribution and fit to 129 observations with 130.23 null deviance. Number of capped brood was estimated using HoneyBeeComplete software. Top four models and the null model are shown.

Model	ΔqAIC	Df	Weight	Residual Deviance
Y ~ S * Yr + Stk	0.0	9	0.784	130.23
Y ~ Stk * Yr + S * Yr	4.14	11	0.099	130.23
Y ~ S + Stk	6.24	6	0.035	130.33
Y ~ Stk + S + Yr	6.96	7	0.024	130.32
Y ~ 1	32.73	2	0.000	188.38

Table S17: Model selection using Akaike information criterion (AIC) for the effect of apiary site (S) (e.g., North, South BK, and South RS), queen stock (Stk) (e.g., BC, HI, and NZ), and sampling year (Yr) (e.g, 2017, and 2018) on mean number of adult bees, (Y), fit with a negative binomial error distribution and fit to 128 observations with 130.48 null deviance. Number of adult bees was estimated using a photo ladder [2]. Top four models and the null model are shown.

Model	ΔAIC	Df	Weight	Residual Deviance
Y ~ S * Stk * Yr	0.0	19	0.996	128.76
Y ~ Stk * Yr + S * Yr	12.62	11	0.002	128.96
Y ~ S * Y + Stk	13.81	9	0.001	129.00
Y ~ Stk * Yr + S * Yr + S * Stk	14.17	15	0.001	128.90
Y ~ 1	121.19	2	0.000	130.48

Table S18: Model selection using Akaike information criterion (AIC) for the effect of apiary site (S) (e.g., North, South BK, and South RS), queen stock (Stk) (e.g., BC, HI, and NZ), and sampling year (Yr) (e.g, fall 2017 and spring 2018) on mean cluster score, (Y), fit with a gaussian error distribution and fit to 141 observations with 685.53 null deviance. Top four models and the null model are shown.

Model	ΔAIC	Df	Weight	Residual Deviance
Y ~ S * Yr + S * Stk	0.0	13	0.757	273.08
Y ~ S * Yr + S * Stk + Stk * Yr	2.62	15	0.205	268.55
Y ~ S * Yr + Stk	7.41	9	0.019	307.85
Y ~ S * Stk * Yr	7.98	19	0.014	259.06
Y ~ 1	105.00	2	0.000	685.53

Table S19: Model selection using Akaike information criterion (AIC) for the effect of apiary site (S) (e.g., North, South BK, and South RS), queen stock (Stk) (e.g., BC, HI, and NZ), and sampling year (Yr) (e.g, 2017, and 2018) on mean honey production, (Y), fit to 125 observations with 118.49 null deviance. Top four models and the null model are shown.

Model	ΔAIC	Df	Weight	Residual Deviance
Y ~ S * Yr	0.0	7	0.640	48.69
Y ~ S * Yr + Stk	1.67	9	0.278	47.56
Y ~ S * Yr + Stk * Yr	4.97	11	0.053	47.00
Y ~ S * Yr + S * Stk	6.52	13	0.025	45.74
Y ~ 1	100.31	2	0.000	118.49

Table S20: General linear hypothesis post-hoc testing on the top model fit selected by Akaike Information Criterion (AIC) for the effect of apiary site (e.g., North, South BK, and South RS) and sampling year (e.g., 2017 and 2018) on honey production. p values were adjusted for Type I error using the Bonferroni method.

**Post-hoc Comparisons**	**Std. Error**	**z value**	**Pr(>|z|)**
**Apiary x Sampling Year Interaction**			
South BK apiary in 2017 - North apiary in 2017	0.17	-5.40	< 0.001
South RS apiary in 2017 - North apiary in 2017	0.18	-10.92	< 0.001
North apiary in 2018 - North apiary in 2017	0.18	-3.98	0.001
South BK apiary in 2018 - North apiary in 2017	0.19	-9.04	< 0.001
South RS apiary in 2018 - North apiary in 2017	0.22	-6.97	< 0.001
South RS apiary in 2017 - South BK apiary in 2017	0.21	-5.09	< 0.001
North apiary in 2018 - South BK apiary in 2017	0.21	1.00	1.000
South BK apiary in 2018 - South BK apiary in 2017	0.22	-3.78	0.002
South RS apiary in 2018 - South BK apiary in 2017	0.24	-2.47	0.205
North apiary in 2018 - South RS apiary in 2017	0.21	5.87	< 0.001
South BK apiary in 2018 - South RS apiary in 2017	0.22	1.02	1.000
South RS apiary in 2018 - South RS apiary in 2017	0.24	1.87	0.919
South BK apiary in 2018 - North apiary in 2018	0.22	-4.57	< 0.001
South RS apiary in 2018 - North apiary in 2018	0.24	-3.24	0.018
South RS apiary in 2018 - South BK apiary in 2018	0.25	0.90	1.000

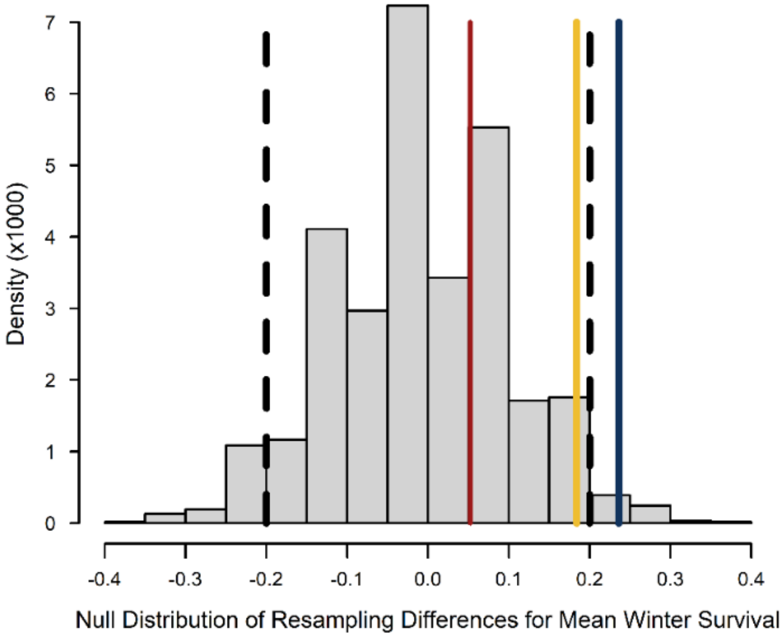

Figure S1. Frequency null distribution of resampling differences of honey bee winter colony survival for colonies headed by three different queen stocks (i.e., British Columbia (BC), Hawaii (HI), and New Zealand (NZ)) with 95% confidence intervals plotted (dashed lines). Observed differences in winter colony survival are plotted for BC-HI, BC-NZ, and Hi-NZ comparisons in yellow, blue, and red, respectively. Any observed treatment comparison line located outside of the null distributions' 95% confidence intervals are significantly different from the null, ($p < 0.05$).

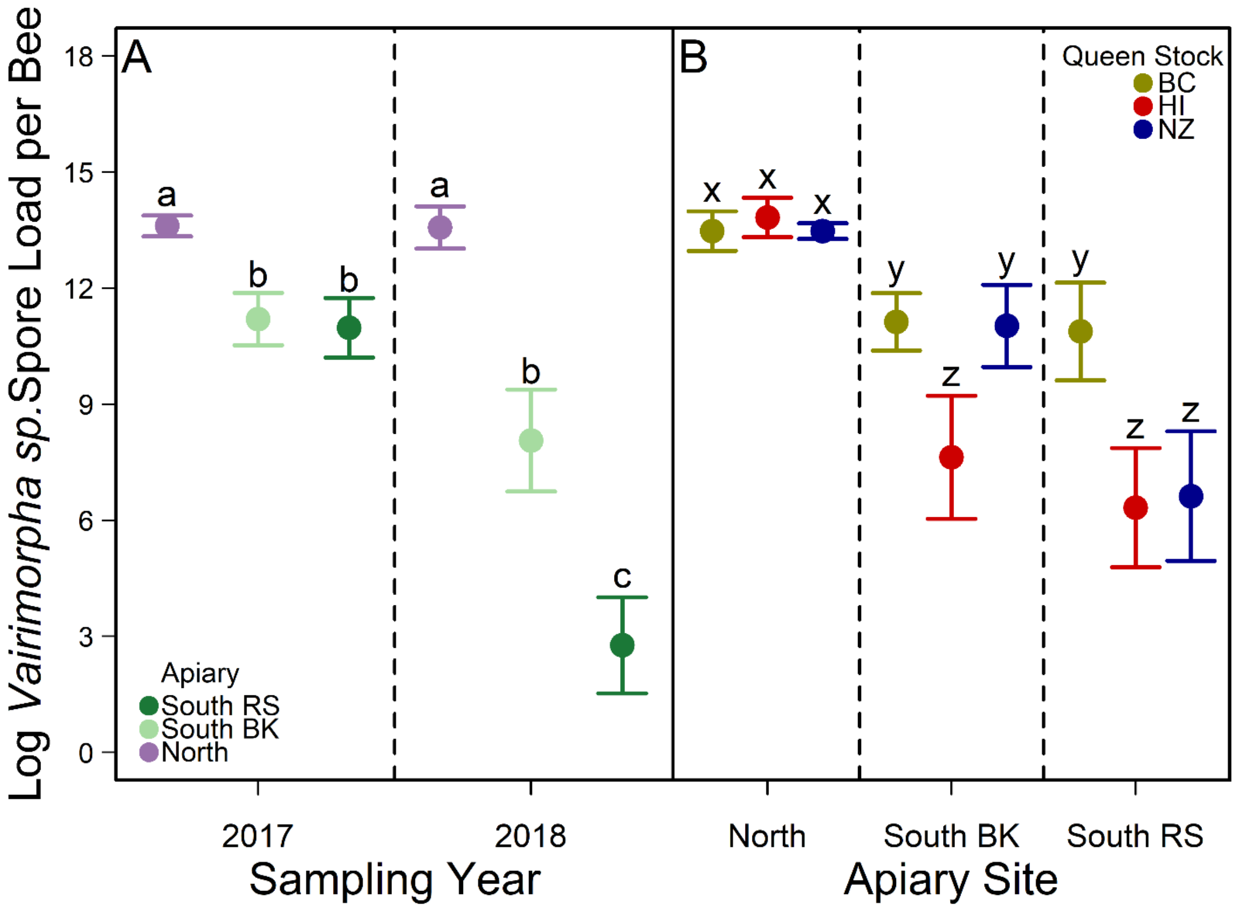

Figure S2. A) Observed mean (± SE) log *Vairimorpha* sp. spore counts assayed in 2017 and 2018 from colonies located at three experimental apiary sites (i.e., North, South BK, and South RS in purple, light green, and dark green, respectively) in Alberta, Canada. Different letters among apiary sites across sampling years indicate significant differences p < 0.05 (Table S8). B) Observed mean (± SE) log *Vairimorpha* sp. spore counts assayed from colonies located at three experimental apiary sites (i.e., North, South BK, and South RS in Alberta, Canada, headed by three queen stocks (i.e., BC, HI, and NZ in yellow, red, and blue, respectively). Different letters among queen stocks across apiary site indicate significant differences p < 0.05 (Table S8).

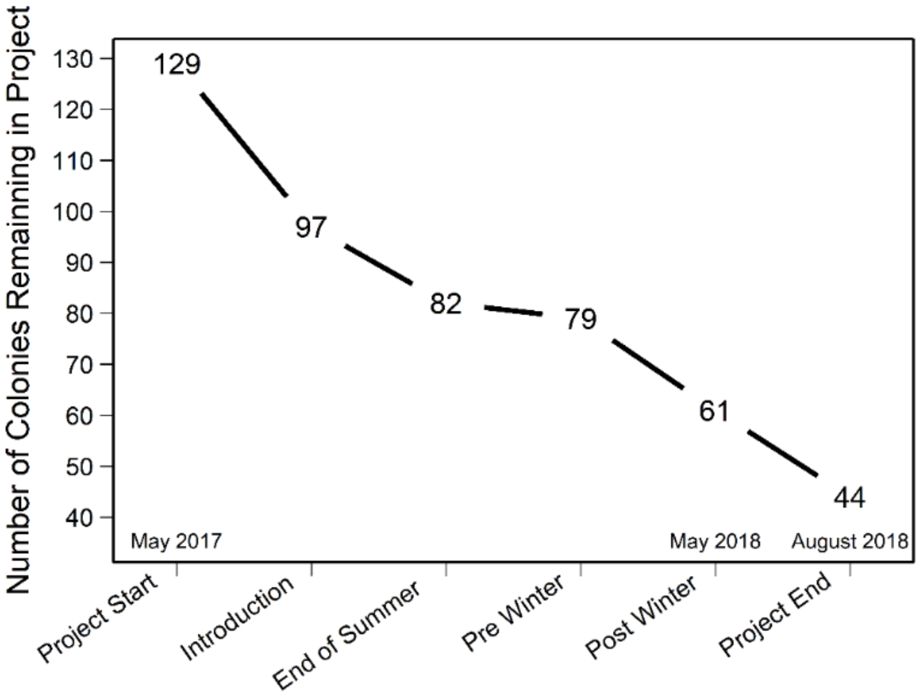

Figure S3. Timeline of colonies remaining in the project between May 2017 and August 2018. Colonies were assessed several times each season and were removed from the project if the original marked queen could not be identified or the colony died (i.e., fewer than four frames of bees).

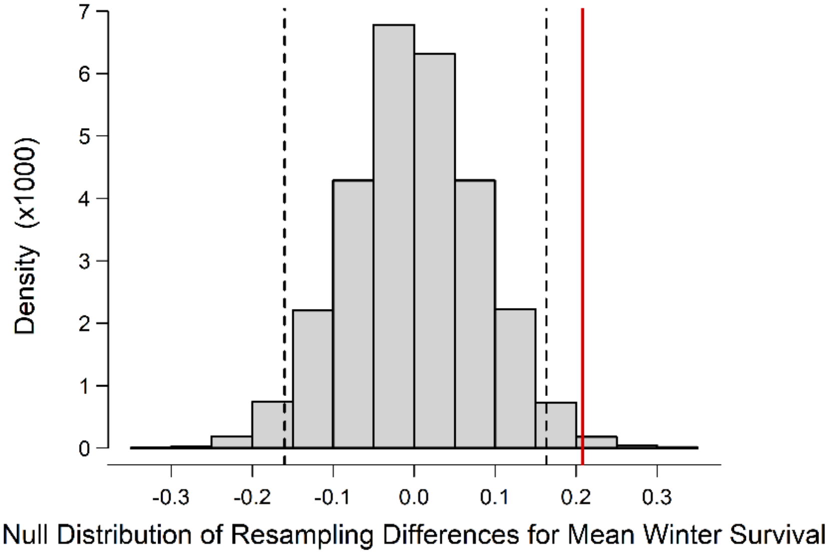

Figure S4. Frequency null distribution of resampling differences of honey bee winter colony survival for colonies headed by either domestic (i.e., British Columbia (BC)) or imported (i.e., Hawaii (HI) and New Zealand (NZ)) queen stocks with 95% confidence intervals plotted (dashed lines). Observed differences in winter colony survival plotted for domestic and imported comparisons in red are located outside of the null distributions' 95% confidence intervals and are therefore significantly different from the null, ($p < 0.05$).

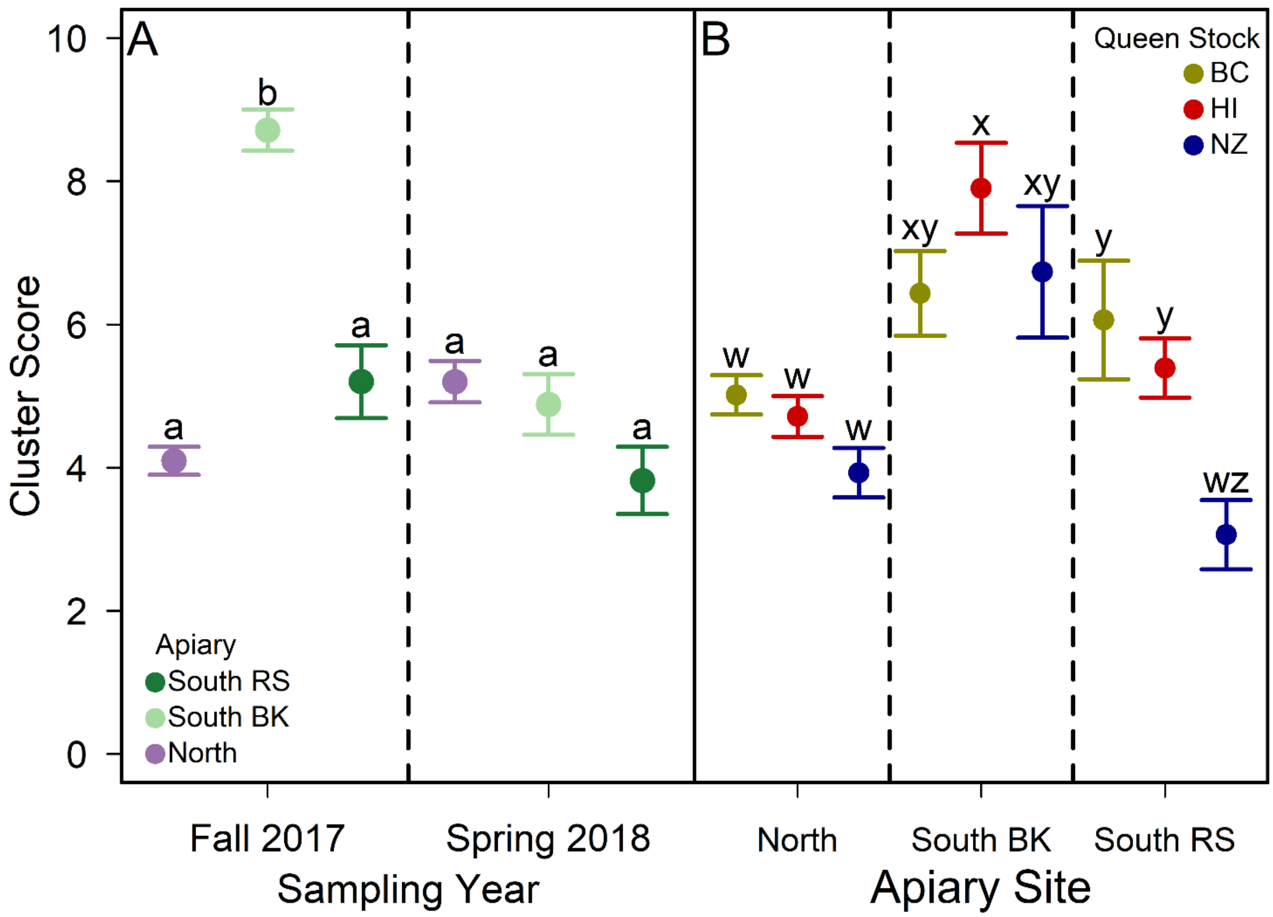

Figure S5. A) Observed mean (± SE) cluster scores assayed in 2017 and 2018 from colonies located at three experimental apiary sites (i.e., North, South BK, and South RS in purple, light green, and dark green, respectively) in Alberta, Canada. Different letters among apiary sites across sampling years indicate significant differences $p < 0.05$ (Table S18). B) Observed mean (± SE) cluster scores assayed from colonies located at three experimental apiary sites (i.e., North, South BK, and South RS in Alberta, Canada, headed by three queen stocks (i.e., BC, HI, and NZ in yellow, red, and blue, respectively). Different letters among queen stocks across apiary site indicate significant differences $p < 0.05$ (Table S18).

REFERENCES

1. CAPA. 2022. Canadian Association of Professional Apiculturists Statement on Honey Bee Wintering Losses in Canada. https://capabees.com/shared/CAPA-Statement-on-Colony-Losses-2021-2022-FV.pdf. (2022). Accessed March 7, 2023.
2. Ovinge, L. P. and Hoover, S. E. Comparison of honey bee (Hymenoptera: Apidae) colony units of different sizes as pollinators of hybrid seed canola. J. Econ. Entomol. **111**, 1535–1541 (2018).
